# Energy competition remodels the metabolic glucose landscape of psoriatic epidermal cells

**DOI:** 10.7150/thno.93764

**Published:** 2024-05-27

**Authors:** Weiwei Liu, Jingwei Jiang, Zeming Li, Yang Xiao, Siyi Zhou, Dehuan Wang, Yi Zou, Tiantian Liu, Ke Li, Huan Liang, Nian'ou Wang, Xiao Xiang, Qiaoli Xie, Rixing Zhan, Jinwei Zhang, Xun Zhou, Li Yang, Cheng-Ming Chuong, Mingxing Lei

**Affiliations:** 1 Key Laboratory of Biorheological Science and Technology of Ministry of Education & 111 Project Laboratory of Biomechanics and Tissue Repair, College of Bioengineering, Chongqing University, Chongqing 400044, China.; 2 Shenzhen Accompany Technology Cooperation, ltd, Shenzhen 518000, China.; 3 State Key Laboratory of Trauma, Burn and Combined Injury, Southwest Hospital, The Third Military Medical University, Chongqing 400038, China.; 4 Department of Dermatology, Chongqing General Hospital, Chongqing 401147, China.; 5 Department of Dermatology and Cosmetology, Chongqing Hospital of Traditional Chinese Medicine, Chongqing 400021, China.; 6 Department of Pathology, Keck School of Medicine, University of Southern California, Los Angeles, California 90033, USA.

**Keywords:** OXPHOS, Glucose metabolism, Cell competition, Disulfidptosis

## Abstract

**Rationale:** Skin cells actively metabolize nutrients to ensure cell proliferation and differentiation. Psoriasis is an immune-disorder-related skin disease with hyperproliferation in epidermal keratinocytes and is increasingly recognized to be associated with metabolic disturbance. However, the metabolic adaptations and underlying mechanisms of epidermal hyperproliferation in psoriatic skin remain largely unknown. Here, we explored the role of metabolic competition in epidermal cell proliferation and differentiation in psoriatic skin.

**Methods:** Bulk- and single-cell RNA-sequencing, spatial transcriptomics, and glucose uptake experiments were used to analyze the metabolic differences in epidermal cells in psoriasis. Functional validation *in vivo* and *in vitro* was done using imiquimod-like mouse models and inflammatory organoid models.

**Results:** We observed the highly proliferative basal cells in psoriasis act as the winners of the metabolic competition to uptake glucose from suprabasal cells. Using single-cell metabolic analysis, we found that the "winner cells" promote OXPHOS pathway upregulation by COX7B and lead to increased ROS through glucose metabolism, thereby promoting the hyperproliferation of basal cells in psoriasis. Also, to prevent toxic damage from ROS, basal cells activate the glutathione metabolic pathway to increase their antioxidant capacity to assist in psoriasis progression. We further found that COX7B promotes psoriasis development by modulating the activity of the PPAR signaling pathway by bulk RNA-seq analysis. We also observed glucose starvation and high expression of SLC7A11 that causes suprabasal cell disulfide stress and affects the actin cytoskeleton, leading to immature differentiation of suprabasal cells in psoriatic skin.

**Conclusion:** Our study demonstrates the essential role of cellular metabolic competition for skin tissue homeostasis.

## Introduction

**Figure 1:**
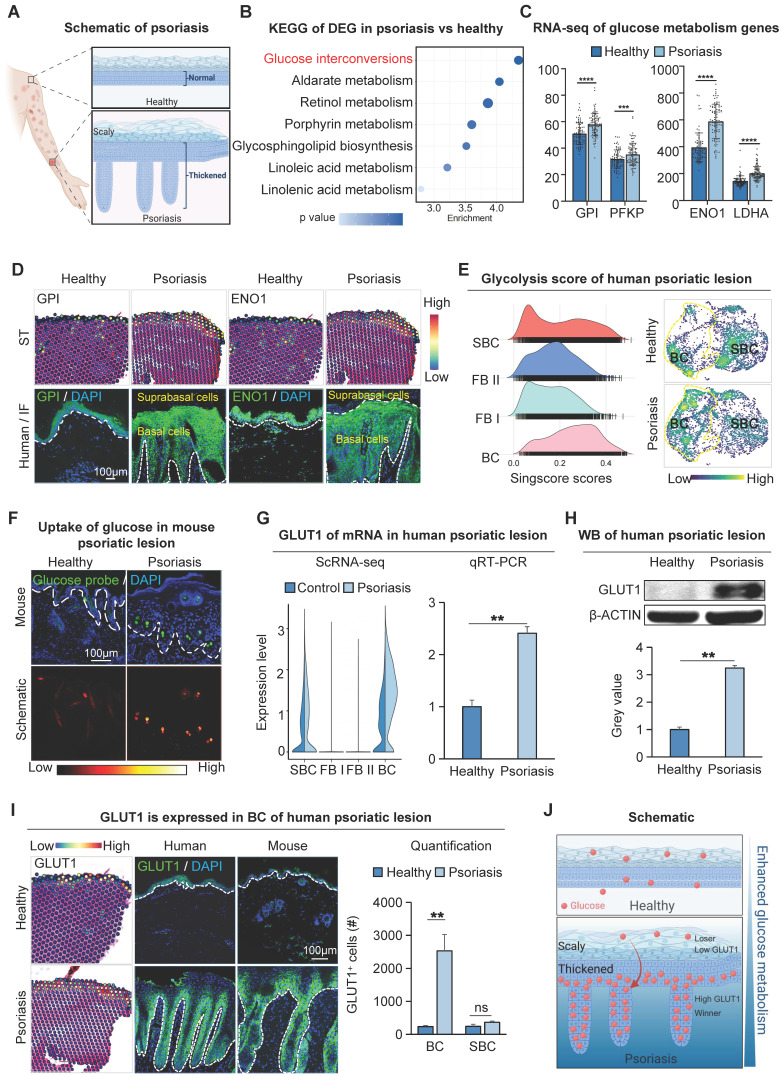
** Increased glucose metabolism in basal cells of psoriatic lesion. A.** Schematic of psoriasis. **B.** KEGG analysis shows the most active glucose metabolism in psoriatic lesions. DEG: differentially expressed genes. **C.** Bulk RNA-seq shows the expression levels of gluconeogenic genes GPI, PFKP, ENO1, and LDHA in human psoriatic lesions. N=82, ****p < 0.0001, ***p < 0.001. **D.** Spatial transcriptome and immunofluorescence staining of glycometabolic genes show that GPI and ENO1 were expressed in the basal cell layer of the human psoriatic lesion. Scale bar, 100 μm. **E.** Single-cell scoring of the glucose metabolism gene set shows that glucose metabolism is active in human psoriatic skin: BC > SBC > FBII > FBI. **F.** Glucose uptake assay shows the strongest glucose uptake in the basal cell layer in the IMQ-induce psoriasis-like mice. Scale bar, 100 μm. **G.** qRT-PCR and single-cell data show that the glucose transporter protein GLUT1 is elevated in human psoriatic skin (right) and is predominantly expressed in the basal cell layer. N=3, **p < 0.01. **H.** Western blot shows expression levels of GLUT1 in human healthy and psoriatic lesions. N=3, **p < 0.01. **I.** Spatial transcriptome and immunofluorescence show the location of GLUT1 expression in human and mouse psoriatic lesion; statistical analysis of GLUT1+ cells. BC: Basal cells. Scale bar, 100 μm. N=3, **p < 0.01, ns, no significant. **J.** Schematic of psoriatic basal cell competition for glucose in suprabasal cells.

Psoriasis is one of the most common chronic immune-mediated skin diseases with an estimated global prevalence of 2-3% 1-3. It is primarily a disease in which the skin barrier is compromised due to the destabilization of cell proliferation and differentiation 4,5. Increasing evidence reveals that psoriasis is related to metabolism disorder 6,7. Abnormalities in amino acid and lipid metabolism were found in psoriasis patients 8-10. Dietary saturated fatty acids can exacerbate skin inflammation in psoriasis 11, and polyamine production in keratinocytes can promote psoriasis progression 12. Nevertheless, in addition to the current medicine with IL-17 inhibitors that treat psoriasis, inhibition of glucose transport in keratin-forming cells appears to be another approach to treating psoriasis 13. Glucose is the main source of energy for mammalian cells, fueling glycolysis and the tricarboxylic acid cycle, and cells possessing a high proliferative capacity have consistently demonstrated a high ability to utilize glucose as a substrate 14. The preferential utilization of glucose in tissues correlates with the phenotype of the cell. For example, increased GLUT1 expression and glucose utilization promote effector T cell function in an asthma model 15,16. The cytological changes in psoriasis are predominantly hyperproliferation of basal epidermal cells which grow downward to form ridges and marked hyperkeratosis (Figure 1A). It is reasonable to speculate this is an energy consumption process.

To ensure tissue fidelity, adaptive cell populations are constantly renewed through proliferation or differentiation, while less-fit cells are actively removed. This renewal is regulated by a surveillance mechanism of cellular competition, which can be observed during the development of organs such as skin, stomach, intestinal tract, and hematopoietic stem cells 17-21. When the epidermis forms a monolayer at the early stages of mouse skin development, the winner cells can kill and engulf the neighboring loser cells 22. In case of an imbalance in skin homeostasis, cells with decreased COL17A1 expression can be cleared by cells with high COL17A1 expression 23,24. During cancer progression, ongoing interactions between cancer and stromal cells lead to cellular metabolic competition 25. These studies suggest that cell competition occurs in maintaining homeostasis of tissue and organs, imbalance of which results in tissue abnormalities or diseases. So, how does energy competition regulate metabolic adaptations in the psoriatic epidermis?

Here, we found that basal epidermal cells compete for glucose in psoriatic skin. Hyperproliferative keratinocytes in psoriasis have superior glucose uptake ability. Basal cells promote oxidative phosphorylation (OXPHOS) and COX7B upregulation through glucose metabolism and promote reactive oxygen species (ROS) production, thereby maintaining keratinocyte hyperproliferation in psoriasis. COX7B promotes psoriasis development by modulating the activity of the PPAR signaling pathway. We also found that SLC7A11 is highly expressed in suprabasal cells that trigger disulfide stress, leading to shrinkage of the suprabasal cell skeleton and the inability to further differentiate, eventually causing hyperkeratosis on the skin surface. Our study suggests that cellular metabolic competition keeps a balance of keratinocyte proliferation and differentiation during psoriasis progression.

## Materials and methods

### Mice

The animal experiment protocol has been approved by the Animal Experiment Ethics Committee of Chongqing University. BALB/c mice (6-8 weeks old male) were purchased from Weitong Lihua Experimental Animal Technology Co., Ltd. (Beijing, China). Mice were housed under the following controlled conditions: a steady temperature of 25 ± 1 °C, a 12 h light/12 h dark cycle 26, with food and water supply. Mice were randomly assigned to experimental groups.

### Inflammatory skin organoid culture

Organoid culture methods for mouse skin organoids can be found in our previously published articles 27-30. Briefly, dorsal skins of newborn mice were floated in 0.25% trypsin (#15050057, Gibco, USA) at 4 °C within 24 h of birth and digested overnight to isolate dermal and epidermal cells. Single epidermal cells were obtained by clipping with scissors, blowing, filtering, and centrifugation. Dermal cells were digested in 0.35% collagenase I (#LS004197, Worthington, USA) at 37 °C for 20 min, then blown, filtered and centrifuged. The isolated dermal and epidermal cells were mixed at a ratio of 1:9 and dropped into the upper chamber of the transwell, and 700 μL of DMEM/F12 (#MT10013CV, Corning, USA) medium containing 10% FBS (#10099-141C, Gibco, USA) was added to the lower chamber. Recombinant proteins of IL22 (#HY-P77969, MCE, China), IL17 (#HY-P700194AF, MCE, China), and TNF-a (#HY-P70800, MCE, China) were added to the medium at a concentration of 2 μg/mL to form an inflammatory organoid model. The cells were cultured in an incubator at 37 °C 5% CO_2_ and the medium was changed every other day. Fluid change: For the next day's change, tilt the 12-well plate, aspirate the old culture fluid from the lower layer, and add 700 μL of fresh culture fluid containing inflammatory factors. Change solution daily. One lance tip treats 1 well to avoid cross-contamination. Three days after changing the inflammatory medium to one containing functionally interfering small molecules, the fluid was changed daily and samples were collected on day 7.

### IMQ-induced psoriasis-like mice and PASI scores

Male BALB/c mice aged 6-8 weeks were topically applied with 62.5 mg 5% IMQ cream (Sichuan Mingxin Pharmaceutical Co., Ltd.) on their shaved back daily for 7 consecutive days 31. To evaluate the severity of skin lesions, the modified Psoriasis Lesion Area and Severity Index (PASI) 32 method was used, including three indicators of erythema, scale, and skin thickness, and scored on a scale of 0 to 4. After 7 days of imiquimod treatment, the lesion samples were collected and immediately fixed in 4% paraformaldehyde solution, or placed at -80 °C for qRT-PCR and western blot assay.

### Baker scores

The histopathological observation of psoriasis mainly adopts the Baker scores, and the specific criteria are as follows: 2.0 points for small Munro abscesses found in the stratum corneum; 0.5 points for hyperkeratosis; 1.0 points for parakeratosis; 1.0 points for thinning or disappearance of the granular layer in the skin layer; Mononuclear or multinucleated cell infiltration in the dermis was scored as 0.5, 1.0, and 1.5 points according to mild, moderate, and severe, respectively; the top of the mastoid was 0.5 points; telangiectasia was 0.5 points 26.

### Small molecules perturbation

After smearing 62.5 mg 5% IMQ on the back skin of mice for 30 min, the mice were subcutaneously injected with 50 μL of GLUT1 inhibitor (Bay-867, 2 mg/kg, MCE), oxidative phosphorylation inhibitor (Rotenone, ADT-OH, 2 mg/kg, MCE), reduced glutathione (GSH), glutathione synthesis inhibitor (L-Buthionine-(S, R)-sulfoximine, 2 mg/kg, MCE) disulfide stress reducing agent (2-Methoxyestradiol, DL-dithiothreitol, 2 mg/kg, MCE), or with the same amount of normal saline as the control group. The mice's skin was injected every day for 7 days, and the samples were collected on the eighth day for H&E and immunofluorescence to observe skin phenotype changes, and qRT-PCR and WB to check the expression changes of related genes and proteins.

### BrdU administration

To label mitotic cells, mice were injected intraperitoneally with BrdU (Beyotime, Sigma) at 50 mg per kg (body weight). Samples were collected 4 h after the intraperitoneal injection followed by BrdU immunofluorescence detection.

### Mitochondrial Membrane Potential Detection Probe

After the dorsal skin of the mouse was harvested, the sample was embedded in a frozen section embedding medium (#SAKURA-4583, Biosharp, China) at -80 °C and cut into 10 μm thick sections with a frozen microtome for probe staining.

For Mito-Tracker Red CMXRos (#C1049B-50μg, Beyotime, China), Prepare 1 mL of the working solution and incubate it at 37 °C for 30 min. Afterward, wash the fixative from the samples with PBS, add the Mito-Tracker Red CMXRos working solution, and incubate at 37 °C for 15-30 min. After discarding the Mito-Tracker Red CMXRos working solution, the samples underwent a 30-minute incubation with DAPI at room temperature. Subsequently, the samples were blocked with an anti-fluorescent extractant, and the red fluorescence intensity of the samples was detected using a laser confocal microscope in the wavelength range of 579-599 nm. Additionally, the first instance of technical term abbreviation was elaborated.

For Enhanced Mitochondrial Membrane Potential Detection Kit (JC-1, C2003S, Beyotime, China), Prepare the JC-1 staining solution. Once the fixative has been washed from the samples, apply the JC-1 working solution. Incubate the samples at 37 °C for 20 min. After incubation, wash the samples twice with JC-1 staining buffer. Apply DAPI for 30 min at room temperature, seal the slices with an anti-fluorescent extractant, and observe them under the laser confocal microscope.

### 2-NBDG Glucose Uptake Fluorescent Probe

On the eighth day of the psoriasis-like mouse model, a 6 mM concentration of 2-NBDG (MX4511, Mokang Biotechnology Co., Ltd., Shanghai) was injected subcutaneously in the imiquimod-coated area on the back of the mice, and after 6 h, the skin on the back of the mice was taken and placed in the direction of sectioning in the embedding cassettes containing frozen sectioning embedding agent, and the embedding agent was rapidly coagulated with liquid nitrogen, and then it was placed in the refrigerator at -80 °C for storage and waiting for sectioning.

The samples were sectioned using a freezer sectioning machine to a thickness of 10 µm. A line was drawn around the sample with an immunohistological paintbrush to prevent spillage of the stain. The samples were gently placed in PBS to remove the cryosection embedding agent, and then 4% PFA was added to the samples to cover the samples and fixed at room temperature for 10 min. The PFA was washed away with PBS, and then DAPI was added to the samples at a dilution ratio and incubated at room temperature for 15 min. The DAPI has washed away, and then the slices were sealed with an antifluorescence quencher and clear nail polish. Finally, the samples were photographed with a Leica laser confocal microscope to observe glucose uptake in mouse skin tissues.

### Glutathione content detection

The skin from mice was collected following 7-day IMQ-induced psoriasis-like mice. The skin was lysed, homogenized in an ice bath, and centrifuged at 8000 g for 10 min at 4 °C. The supernatant was stored for measurement at 4 °C. The measurement tube, standard tube, and blank tube were arranged according to instructions. Add the corresponding reagents to each tube. Finally, the spectrophotometer was preheated for over 30 min. The wavelength was adjusted to 412 nm, and the absorbance was zeroed with distilled water. The glutathione content in the samples was then computed using the appropriate formula.

### Transcriptome analysis

Transcriptome data on human psoriasis was obtained from the GEO database (GSE 54456) for analysis. DESeq2 was used to perform a cluster analysis on the transcriptome data to identify genes that were differentially expressed between psoriatic skin and healthy skin tissue of humans. A threshold of false discovery rate < 0.05 and log2 fold change > 2 was applied to determine significant differences in gene expression. Gene ontology (GO) and Kyoto Encyclopedia of Genes and Genomes (KEGG) enrichment analysis were conducted on genes showing significantly upregulated expression between the two groups using the online data analysis and visualization platform, https://www.bioinformatics.com.cn. Technical term abbreviations are clarified upon first use. The language remains formal, concise, and objective.

The scRNA-seq data for human psoriasis whole skin (GSE162183) was downloaded from the GEO database 33-35. Technical term abbreviations are explained upon first use. We reanalyzed data from both healthy and psoriatic full-thickness skin samples using the statistical software R (version 4.2.0). We implemented the QC, cell screening, normalization, identification of hypervariable genes, and linear dimensionality reduction using the R package Seurat version 4.1.3. Afterward, we referred to the article's cell marker genes for group annotation and complete data preprocessing. For further enrichment analysis of metabolic pathways, we utilized the R package scMetabolism version 0.2.1 and the single-cell gene set scoring method.

The spatial transcriptome data for human psoriasis whole skin (GSE225475) was downloaded from the GEO database. We reanalyzed data from both healthy and psoriatic full-thickness skin samples using the statistical software R (version 4.2.0).

### Statistical analyses

All experimental data were from three independent repeated experiments, expressed as mean ± standard deviation (SD). All statistical analyses, including one-way analysis of variance (ANOVA), multiple comparisons, and t-tests were performed using GraphPad Prism 9 software (GraphPad Software Inc., version 9.0.0). (*) P < 0.05, (**) P < 0.01, (***) P < 0.001, and (****) P < 0.0001 were considered as the threshold for statistical significance.

## Results

### Increased glucose metabolism within the basal epidermal cells of the human psoriatic lesion

To examine the metabolic changes in psoriatic skin cells, we performed KEGG enrichment analysis on the up-regulated differential genes of human full-thickness skin bulk RNA-sequencing (RNA-seq) data from psoriasis and healthy groups. The results showed that glucuronate interconversion pathways were significantly enriched (Figure 1B). RNA-seq data showed that the expression of GPI, PFKP, ENO1, and LDHA related to the glucose metabolism pathway was significantly increased in the human psoriasis group compared to the control (Figure 1C). To examine the spatial localization of these genes, we scrutinized spatial transcriptome (ST) data of healthy and psoriatic human whole-layer skin 36. We performed dimensionality reduction clustering and utilized KLK7 and COL17A1 to demonstrate the spatial position of basal and suprabasal cells (Figure S1A). ST analysis and immunofluorescence staining showed that glycogen genes were more highly expressed in healthy skin basal cells and suprabasal cells. However, in human psoriatic skin, glucose metabolism genes such as GPI, ENO1, etc. are predominantly expressed in proliferating basal cells compared to the suprabasal cells (Figure 1D and S1B).

To further confirm the active glucose metabolism in basal cells of psoriatic skin, we analyzed single-cell sequencing (scRNA-seq) data from both healthy and psoriatic human skin 33, and scaled-down and annotated cell subpopulations in the epidermis and dermis separately. Epidermal cells were separated into two subclusters (BC, SBC), and dermal cells into two subclusters (FB I, FB II) (Figure S1C-D). The result showed that glucose metabolism genes GPI, PFKF, ENO1, and LDHA were increased in the human psoriasis group and predominantly expressed in the basal cell subpopulation (Figure S1E). We then performed single-cell metabolic scoring of the different subpopulations to show glucose metabolic activities in different subpopulations by the strength of the glycolytic pathway. We observed a more active glycometabolism in the epidermal cell population compared to the dermal cell population, and a higher glycometabolism score in the basal cells compared to the suprabasal cells (Figure 1E). Thus, the glycometabolic activity is basal cell > suprabasal cells > dermal cells. These results suggest that glucose metabolism is more active in psoriatic skin cells compared to the healthy skin, and is more active in hyperproliferative basal cells compared to suprabasal cells.

### Increased expression of GLUT1 provokes glucose uptake in basal cells of psoriatic lesions

To directly detect the glucose uptake capacity of skin cells, we intracutaneously injected the glucose uptake fluorescent probe 2-NBDG into the mouse dorsal skin and observed enhanced glucose uptake by basal cells in the psoriasis group compared to the healthy group (Figure 1F). It has been reported that glucose transporter proteins regulate the uptake of glucose in most tissues. To explore the key genes mediating glucose uptake in psoriasis, we performed qRT-PCR and transcriptome analyses of genes from glucose transporter protein families 1-14. The results showed that GLUT1 was predominantly increased in basal cells in the psoriasis group compared to healthy individuals and controls (Figure 1G and S1F-H). This was also verified by qRT-PCR and western blotting in human psoriatic skin (Figure 1G-H). Immunofluorescence staining and ST analysis showed that the expression of GLUT1 is significantly increased in the basal cells in the human psoriasis group compared to the healthy group (Figure 1I), consistent with the location of active gluconeogenic pathway expression (Figure 1E). Therefore, we propose that the increased GLUT1 expression and enhanced glucose uptake in basal cells aggravate psoriasis progression. Therefore, hyperproliferating basal cells with high metabolic fitness may take up glucose from surrounding cells and lead to a state of cellular competition in which cells compete with each other for the limited glucose metabolic substrates available in the environment. (Figure 1J).

**Figure 2:**
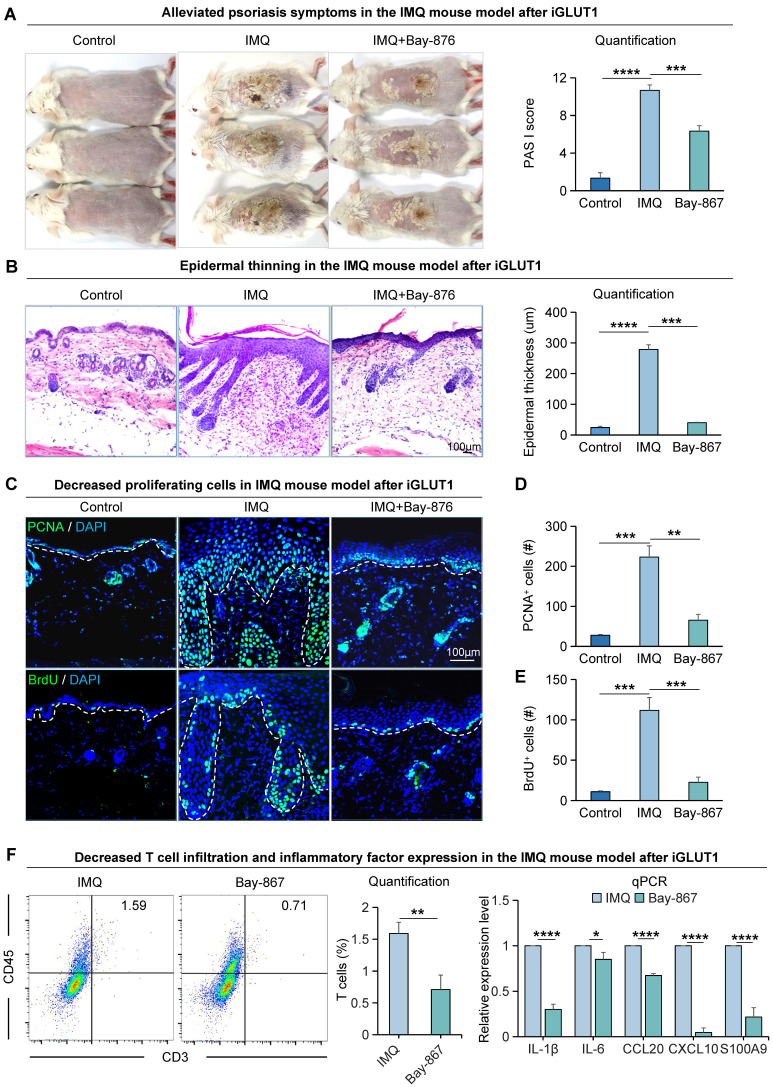
** The proliferative ability of epidermal cells in the IMQ-induced psoriasis-like mice is attenuated after Bay-867 treatment. A.** Macroscopic features and PASI scores of the back of mice in control, IMQ, and IMQ+Bay-867 groups. iGLUT1: inhibition of GLUT1 expression. Bay-867: inhibitor of GLUT1. N=3, ****p < 0.0001, ***p < 0.001. **B.** Statistical analysis of representative H&E staining and epidermal thickness on the back of mice in control, IMQ, and IMQ+Bay-867 groups. Scale bar, 100 μm. N=3, ****p < 0.0001, ***p < 0.001. **C.** Immunofluorescence staining of PCNA and BrdU shows a reduction of proliferating cells in the IMQ mouse model after Bay-867 treatment. Scale bar, 100 μm. **D.** Statistical analysis of PCNA+ cells in control, IMQ, and IMQ+Bay-867 groups. N=3, ****p < 0.0001, **p < 0.01. **E.** Statistical analysis of BrdU+ cells in control, IMQ, and IMQ+Bay-867 groups. N=3, ****p < 0.0001. **F.** Flow cytometry analysis of T cell infiltration after iGLUT1 (left), and qRT-PCR showing expression levels of psoriasisassociated inflammatory factors after iGLUT1 (right). N=3, ****p < 0.0001, **p < 0.01, *p < 0.05.

To explore the impact of GLUT1 on the progression of psoriasis, we utilized a psoriasis-like mice model induced by imiquimod (IMQ) 37 (Figure S2A). We found that mice treated with GLUT1 inhibitor (BAY-867) had a milder psoriasis-like skin phenotype compared to the psoriasis-like mice model induced by IMQ (Figure 2A). PASI scores were significantly reduced in the GLUT1-inhibited group compared to the IMQ-treated group (Figure 2A). H&E staining showed a thinner skin epidermis, fewer downwardly extending ridges, and lower baker scores in the GLUT1-inhibited group compared to the IMQ-treated group (Figure 2B, S2B). qPCR results showed increased production and secretion of psoriasis-associated factors in the IMQ-treated group, including pro-inflammatory cytokines (IL-1β and IL-6), chemokines (CCL20 and CXCL10), and antimicrobial peptides (S100A9) (Figure S2C). Immunofluorescence staining and statistical analysis showed that the number of PCNA^+^, BrdU^+^, and P63^+^cells as the markers of proliferating cells, was significantly decreased in the GLUT1-inhibited group compared to the IMQ-treated group (Figure 2C-E, S2D-E). Expression of E-cadherin and K14, marker genes for epithelial cells, is also decreased after inhibition of GLUT1 (Figure S2D, F-G). Basal cells and suprabasal cells marker genes COL17A1, P-cadherin, and K16 exhibit a decrease in their expression post the inhibition of GLUT1 (Figure S2D, H-J). Flow cytometry analysis showed reduced T-cell infiltration in the GLUT1-inhibited group compared to the IMQ-treated group, and qPCR results showed reduced production and secretion of psoriasis-associated factors in the GLUT1-inhibited group compared to the IMQ-treated group (Figure 2F). These suggest that after GLUT1 inhibition, the epidermis becomes thinner compared to IMQ-induced psoriasis-like mice. These results indicate that GLUT1 can reduce the hyperproliferation of epidermal cells in psoriasis.

### OXPHOS is highly expressed in basal cells in psoriatic lesions

**Figure 3:**
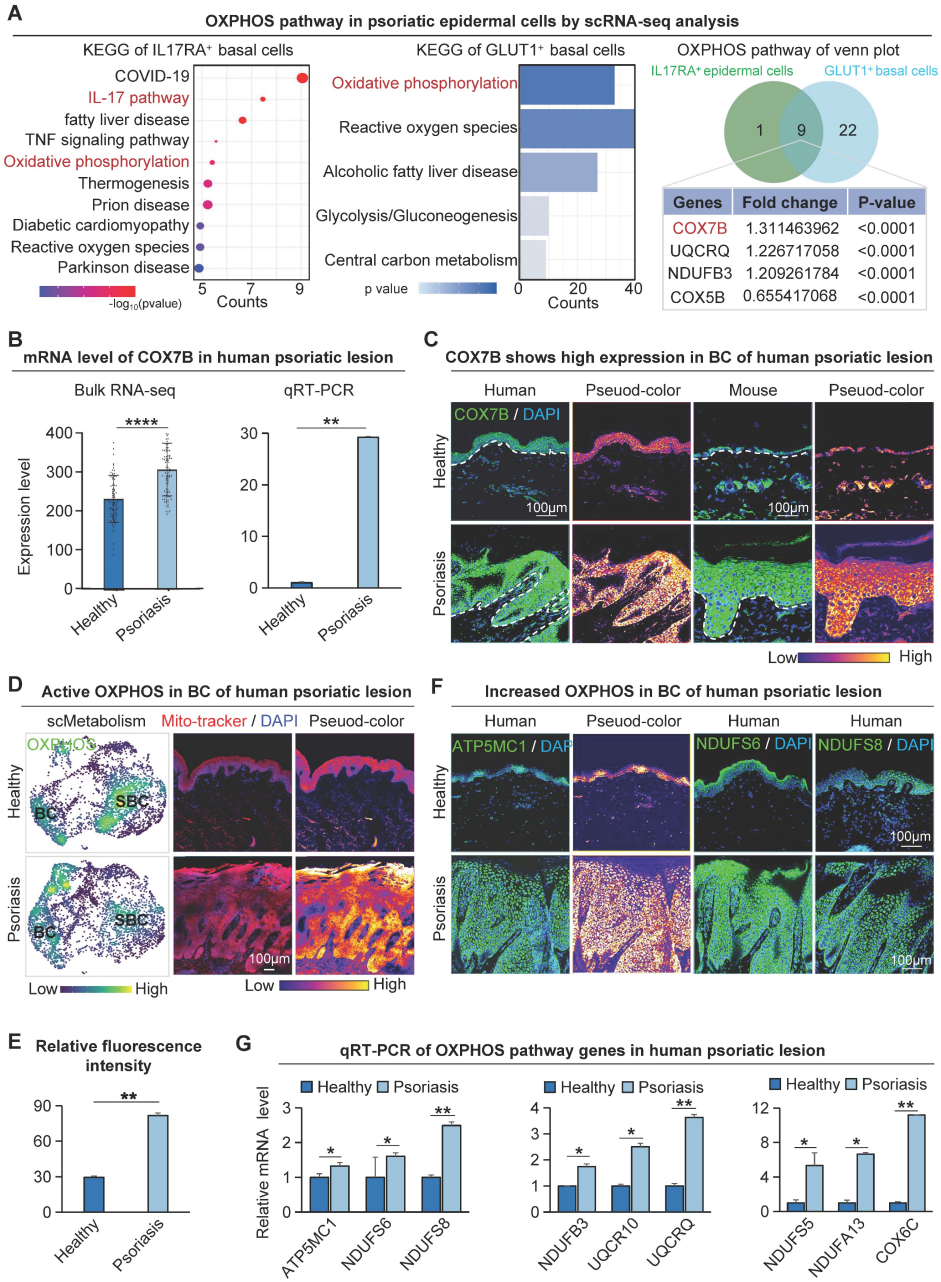
Glucose metabolism pr omotes COX7B-mediated upr egulation of OXPHOS in psor iatic basal cells. **A.** KEGG analysis of IL17RA+ basal cell differential genes in human healthy and psoriatic skin; Differential gene KEGG enrichment analysis of GLUT1+ basal cells in the healthy and psoriatic lesion (left); Venn diagram shows gene overlap between IL17RA+ OXPHOS and GLUT1+ OXPHOS (middle); List of overlapping genes (right). **B.** Bulk RNA-seq and qRT-PCR of the human psoriatic lesion shows gene expression levels of COX7B. N=82, ***p < 0.001, **p < 0.01. **C.** Immunofluorescence staining of COX7B shows its expression in the basal cell layer of the human psoriatic lesion. Scale bar, 100 μm. **D.** Single-cell metabolic scoring of the OXPHOS gene set; Mitochondrial probes detect OXPHOS activity in human skin basal cells. Scale bar, 100 μm. **E.** Statistical analysis of fluorescence intensity of mitochondrial probe for detection of OXPHOS activity in human skin basal cells. N=3, **p < 0.01. **F.** Immunofluorescence staining shows the expression location of the OXPHOS pathway genes ATP5MC1, NDUFS6, and NDUFS8 in human psoriatic skin. Scale bar, 100 μm. **G.** qRT-PCR shows the expression level of OXPHOS genes in human psoriatic lesions. N=3, **p < 0.01.

Glucose is the primary energy source for mammalian cells, fueling glycolysis and the tricarboxylic acid cycle 38,39 (Figure S3A). Since IL17 is an important cytokine mediating psoriasis development, to investigate the relationship between IL17 and keratinocyte metabolism, we conducted KEGG enrichment analysis using differential genes from IL17RA^+^ basal cells in healthy and psoriatic conditions. The results indicate a significant enrichment of oxidative phosphorylation in epidermal cells (Figure 3A). Meanwhile, to clarify the specific metabolic molecular mechanisms that drive glucose metabolism in metabolically competitive basal cells (“winner cells”) in psoriasis, we extracted the differentially expressed genes in GLUT1^+^ vs GLUT1^-^ human basal cells from scRNA-seq of both healthy and psoriatic datasets for KEGG enrichment analysis. The results showed that the oxidative phosphorylation metabolic pathway was significantly enriched (Figure 3A). The fold change of COX7B ranks first among the genes involved in the two KEGG-enriched pathways, suggesting that COX7B may be a key target for energy metabolism in basal cells during psoriasis progression. RNA-seq analysis and qRT-PCR (Figure 3B) showed that the mRNA expression of COX7B was significantly increased in the human psoriatic lesion compared to the healthy skin. Western blot (Figure S3B) and immunostaining (Figure 3C) confirmed that the protein level of COX7B was significantly increased in the human psoriatic lesion compared to the healthy skin, particularly in the hyperproliferative basal cell layer.

To further clarify where oxidative phosphorylation metabolism is present in psoriasis, we did a single-cell metabolic gene set scoring for the oxidative phosphorylation pathway, which showed significant enrichment in the basal cell layer of the psoriasis group (Figure 3D). We also used a mitochondrial fluorescent probe to characterize the oxidative phosphorylation activity in human skin tissue (Figure 3D-E). Consistent with the single-cell metabolic analysis, the results showed that the oxidative phosphorylation pathway was more active in the basal cells than in the suprabasal cells. In addition to COX7B, western blot, bulk RNA-seq, scRNA-seq, and ST analysis data showed that other oxidative phosphorylation pathway-related genes ATP5MC1, NDUFS6, and NDUFS8 were also increased in the human psoriasis group and predominantly expressed in the basal layer (Figure S3B-E). Immunofluorescence staining showed that the oxidative phosphorylation pathway genes were mainly expressed in basal cells of the human psoriasis group (Figure 3F). qRT-PCR showed that the expression of oxidative phosphorylation pathway genes was significantly up-regulated in the human psoriasis group compared to the healthy group (Figure 3G). The qRT-PCR results showed reduced expression of genes associated with OXPHOS in the GLUT1-inhibited group compared with the IMQ-treated group (Figure S3F).

To illustrate that oxidative phosphorylation metabolism is most active in basal cells, KEGG enrichment analyses were performed using differentially expressed genes in suprabasal cells from the human psoriasis group and control, or in all epidermal cells. The results showed that none of the oxidative phosphorylation metabolic pathways were significantly enriched (Figure S3G). These results suggest that basal cells in psoriasis are mainly supplied with energy through the glucose-oxidative phosphorylation pathway.

Enrichment analysis was performed to identify differentially expressed genes in GLUT1^+^ basal cells from healthy and psoriatic individuals and identified significant pathways including the regulation of ATP synthase activity and antioxidant activity pathways (Figure S3H). The above results indicate that the oxidative phosphorylation pathway is present in the metabolically active basal cells, which is consistent with the GLUT1 expression in the skin tissues of psoriasis. This suggests that the glucose metabolism (GLUT1)-oxidative phosphorylation metabolism (COX7B) axis may promote the hyperproliferation of basal cells.

### Suppression of energy metabolism alleviates psoriasis symptoms

**Figure 4:**
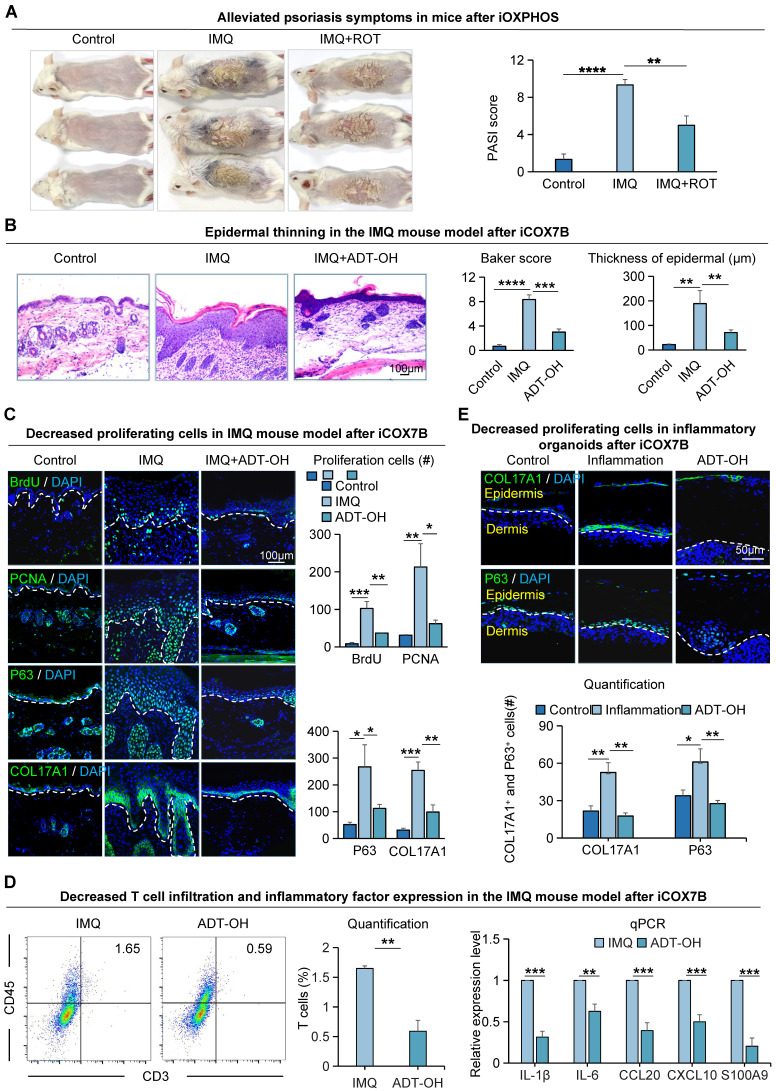
The pr olifer ative ability of epider mal cells in IMQ-induced psor iasis-like mice is attenuated after Rotenone treatment. **A.** Macroscopic feature and PASI scores of the back of mice in control, IMQ, and IMQ+ROT groups. iOXPHOS: inhibition of OXPHOS. ROT: Rotenone, inhibitor of OXPHOS. N=3, ****p < 0.0001, **p < 0.01. **B.** Representative H&E staining and baker score of the back of mice in control, IMQ, and IMQ+ADT-OH groups; Statistical analysis of epidermal thickness. Scale bar, 100 μm. N=6, ****p < 0.0001, **p < 0.01. **C.** Immunofluorescence staining of BrdU, PCNA, P63, and Col17a1 shows cell proliferation in IMQ mice after ADT-OH treatment; Statistical analysis of BrdU+ cells, PCNA+ cells, P63+ cells, and Col17a1+ cells. Scale bar, 100 μm. N=3, ***p < 0.001, **p < 0.01, *p < 0.05. **D.** Flow cytometry analysis of T cell infiltration after iCOX7B (left), and qRT-PCR showing expression levels of psoriasisassociated inflammatory factors after iCOX7B (right). N=3, ***p < 0.001, **p < 0.01, *p < 0.05. **E.** Immunofluorescence staining of COL17A1 and P63 in an organoid model of control, inflammation, and ADT-OH groups. ADT-OH: inhibitor of COX7B. Scale bar, 50 μm.

To investigate the effects of COX7B on psoriasis progression, we did functional perturbation on the OXPHOS pathway. We observed that mice treated with COX7B inhibitors (ADT-OH) had a milder psoriasis-like phenotype compared to the IMQ-induced psoriasis-like mice, and the PASI scores were significantly reduced in the rotenone treat-mice compared to the psoriasis-like mice (Figure 4A). H&E staining showed a thinner epidermis, fewer downward extending ridges, and lower baker's score in the ADT-OH-treated mice and rotenone-treated mice compared with the psoriasis-like mice (Figure 4B, S4A). Immunofluorescence staining showed that the number of BrdU^+^, PCNA^+^, P63^+^, and COL17A1^+^ cells was significantly decreased in the ADT-OH-treated skin compared to the psoriasis-like mice (Figure 4C). The expression of K14, E-cadherin, P-cadherin, and K16, marker gene of epithelial cells, was decreased in the ADT-OH-treated mice compared with the psoriasis-like mice, suggesting a decrease in epidermal thickness (Figure S4B**)**. Flow cytometry analysis showed reduced T-cell infiltration in the ADT-OH-treated group compared to the IMQ-treated group, and qPCR results showed reduced production and secretion of psoriasis-associated factors in the ADT-OH-treated group compared to the IMQ-treated group (Figure 4D). Functional perturbation of the oxidative phosphorylation pathway using rotenone also validated these results (Figure S4C).

To gain insight into the impact of oxidative phosphorylation metabolism on inflammatory cutaneous cells, we created a mouse organoid culture model in which epidermal cells, dermal cells, and immune cells were included (Figure S4D) 27. Double staining of K14 and Vimentin showed that the inflammatory organoid had intact dermal and epidermal structures (Figure S4E). Treatment with inflammatory cytokines dramatically increased the production and secretion of psoriasis-related factors in inflammatory skin organoids, including proinflammatory cytokines (IL-1β and IL-6), chemoattractant proteins (CCL20 and CXCL10), and antimicrobial peptide S100A9 (Figure S4F). Immunofluorescence and statistical analyses indicated a reduction in the expression of COL17A1 and P63 in organoids following administration of the ADT-OH-treated group (Figure 4E), consistent with the *in vivo* results. K14 and K16 immunostaining showed that the differentiated keratin is increased in the ADT-OH-treated group of organoid cultures (Figure S4G). These results suggest that inhibition of OXPHOS attenuates epidermal cell hyperproliferation in psoriasis-like mice.

**Figure 5:**
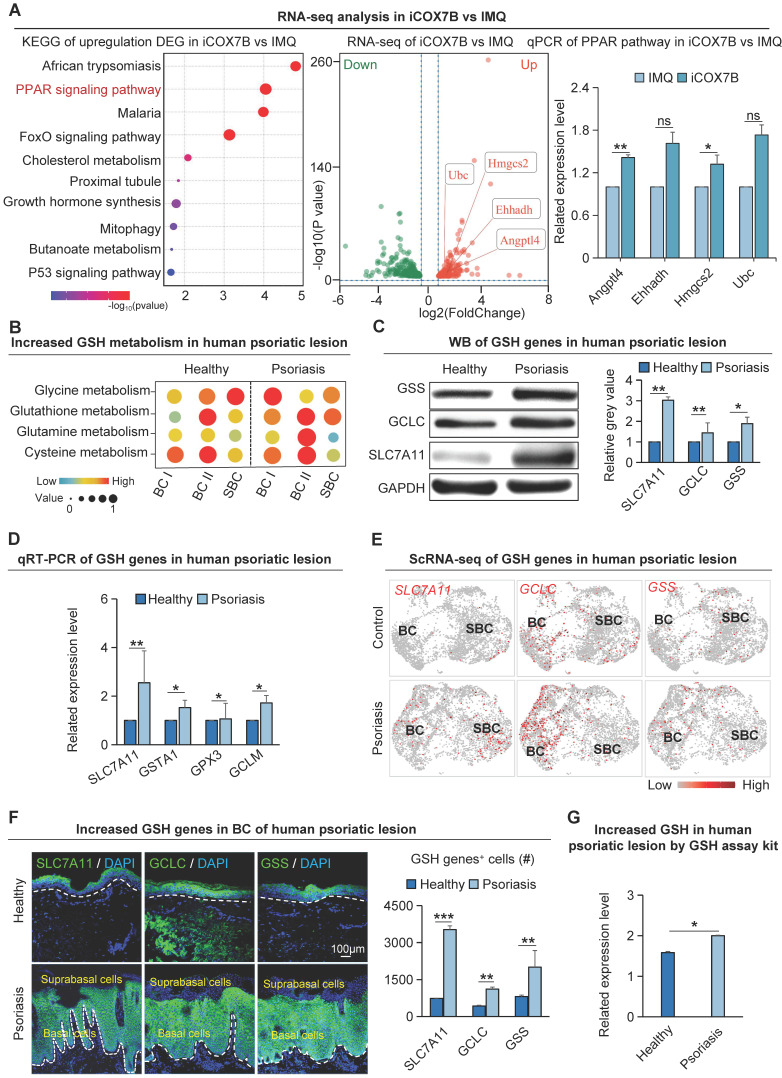
Glutathione metabolism is significantly up-r egulated in basal cells of psor iasis. **A.** Bulk RNA-seq analysis of the downstream pathway of COX7B (left); qRT-PCR showed that the expression levels of PPAR pathway-related bases were reduced after iCOX7B (right). N=3, **p<0.01, *p<0.05, ns, no significant. **B.** Single-cell metabolic analysis of epidermal cells from human healthy and psoriatic lesion; GSH: Glutathione. **C.** Western blot shows protein expression levels of glutathione metabolism genes SLC7A11, GCLC, and GSS in human psoriatic lesions. N=3, **p < 0.01, *p < 0.05. **D.** qRT-PCR shows the expression levels of glutathione metabolism genes in human psoriatic lesions. N=3, **p < 0.01, *p < 0.05. **E.** ScRNA-seq analysis shows the elevated expression of SLC7A11, GCLC, and GSS in the human psoriatic lesions. N=3, **p < 0.01, *p < 0.05. **F.** Immunofluorescence staining of SLC7A11, GCLC, and GSS shows the location of their expression in human psoriatic lesions; GSH+ cells were statistically analyzed. scale bar, 100 μm. N=3, ***p < 0.001, **p < 0.01. **G.** Quantitative analysis of glutathione content in human healthy and psoriatic skin. N=3, *p < 0.05.

To investigate the specific molecular mechanisms by which COX7B promotes psoriasis progression, transcriptome sequencing was performed on psoriasis-like mouse samples with suppressed COX7B expression in this study. The results showed that the peroxisome proliferator-activated receptor (PPAR) signaling pathway was significantly enriched by KEGG enrichment analysis of up-regulated genes after inhibition of COX7B (Figure 5A). The qRT-PCR results showed that the expression of all the relevant genes of the PPAR signaling pathway was significantly elevated after inhibition of COX7B, and bulk RNA-seq showed the same results (Figure S5A). The above results suggest that COX7B promote psoriasis progression by modulating PPAR signaling pathway activity.

### Mitochondrial hyperrespiration activates glutathione metabolism in psoriasis

A considerable amount of ROS is produced due to the excessive proliferation of psoriatic skin cells. To prevent the toxic damage caused by ROS, the cells activate the glutathione metabolic pathway to maintain redox homeostasis and promote the progression of psoriasis 40. KEGG enrichment analysis on differentially expressed genes in GLUT1^+^ human basal epidermal cells from both healthy and psoriatic datasets revealed that ROS is significantly enriched in the GLUT1^+^ cells (Figure 3A). Single-cell metabolism analysis verified that the glutathione-related anabolic pathways such as glutathione metabolism, cysteine metabolism, glycine metabolism, glutamine metabolism, etc. were significantly upregulated in the human psoriasis group compared to the control (Figure 5B). Moreover, glutathione-related metabolism was more significantly upregulated in the basal layer in the psoriasis group compared with the healthy group (Figure 5B). qRT-PCR and western blot showed that glutathione synthesis genes such as SLC7A11, GCLC and GSS were significantly upregulated in the human psoriasis group compared to the control (Figure 5C-D). To determine if the expression sites of genes associated with glutathione metabolism align with the expression sites of active genes related to oxidative phosphorylation metabolism, we performed scRNA-seq analysis and found that glutathione synthesis-related genes SLC7A11, GCLC, and GSS were significantly upregulated in the basal cells of the human psoriasis group compared to the control (Figure 5E). Immunofluorescence staining showed that the expression of SLC7A11, GCLC, and GSS was mainly increased in the basal cells of human psoriasis group compared with the control (Figure 5F). Skin tissues in the IMQ-induced psoriasis-like mouse model demonstrated a comparable pattern of expression (Figure S5B-C) Glutathione content assay revealed significant upregulation of GSH content in the IMQ-induced psoriasis-like lesion compared to control (Figure 5G). The qRT-PCR results showed reduced expression of genes related to glutathione synthesis after inhibition of OXPHOS metabolism (Figure S5D). The above results indicate that glutathione is mainly upregulated in the basal cells of psoriatic skin. In the process of energy metabolism-promoted cell hyperproliferation, glutathione may balance the cellular redox state and keep the pathological proliferation of psoriatic skin cells.

### Modulation of glutathione metabolism regulates psoriasis progression

**Figure 6:**
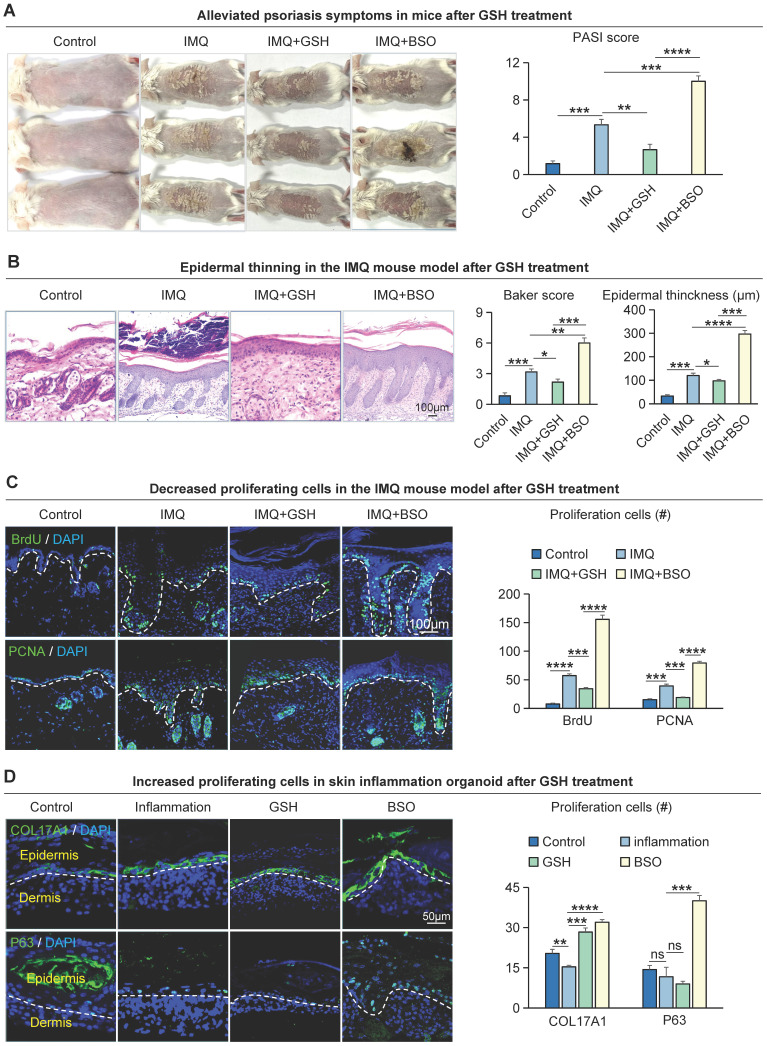
Glutathione metabolism r egulates the pr olifer ation of epider mal cells in IMQ-induced psor iasis-like mice. **A.** Macroscopic feature and PASI scores of the back of mice in control, IMQ, IMQ+GSH, and IMQ+BS0 groups. GSH: glutathione; BSO: buthionine sulfoximine, inhibitor of glutamylcysteine synthetase biosynthesis. N=3, ****p < 0.0001, ***p < 0.001, **p < 0.01. **B.** Statistical analysis of representative H&E staining, baker score, and epidermal thickness of the back of mice in control, IMQ, IMQ+GSH, and IMQ+BSO groups. Scale bar, 100 μm. N=3, ****p < 0.0001, ***p < 0.001, **p < 0.01, *p < 0.05. **C.** Immunofluorescence staining of BrdU, and PCNA shows proliferation of epidermal cells in IMQ mouse model. Scale bar, 100 μm. N=3, ****p < 0.0001, ***p < 0.001. **D.** Immunofluorescence staining of COL17A1 and P63 and statistical analysis of COL17A1+ cells and P63+ cells in organoid models of control, inflammation, GSH, and BSO groups. Scale bar, 50 μm. N=3, ****p < 0.0001, ***p < 0.001, **p < 0.01, *p < 0.01, ns no significant.

To investigate the effect of glutathione metabolism on basal cell proliferation in psoriasis, we intracutaneously injected Reduced Glutathione synthesis inhibitors (BSO) or GSH peptides into the dorsal skin of IMQ-induced psoriasis-like mouse. We found that IMQ + BSO-treated group had a more severe psoriasis-like phenotype and a significantly higher PASI scores compared to IMQ-treated group (Figure 6A). In contrast, the GSH peptide-treated group had a milder psoriasis-like phenotype and a reduced PASI scores compared to the IMQ-treated group and IMQ + BSO-treated group (Figure 6A). H&E staining showed that the epidermis of the IMQ + BSO-treated group was thickened, with increased downward-extending ridges and a higher baker's scores, compared to the IMQ-treated group (Figure 6B). Whereas the IMQ + GSH-treated group displayed a reduced epidermal thickness, fewer downward-extending ridges, and lower Baker scores, compared to the IMQ-treated group and IMQ + BSO-treated group (Figure 6B).

To examine the effect of GSH on cell proliferation, we performed immunofluorescence staining for BrdU^+^, PCNA^+^, and KI67^+^ in these mouse skin samples. The result showed that the number of proliferating cells was significantly increased in the IMQ + BSO-treated group, compared to the IMQ- or IMQ + GSH-treated groups (Figure 6C, S6A). The expression of K14, COL17A1, and K16 is decreased in the IMQ+GSH-treated groups and increased in the IMQ + BSO-treated groups (Figure S6B). Immunofluorescence and statistical analyses showed that the expression of COL17A1 and P63 was reduced in the IMQ + GSH-treated group, and was increased in the IMQ + BSO-treated group in organoid culture (Figure 6D), consistent with the *in vivo* results. Epidermal proliferation is significantly decreased in the IMQ + GSH-treated groups but increased in the IMQ + BSO-treated groups (Figure S6C). These results suggest that GSH can negatively regulate the proliferation of epidermal cells in psoriasis-like mice.

### Metabolic reprogramming of epidermal cells regulates the immature differentiation of suprabasal cells

**Figure 7:**
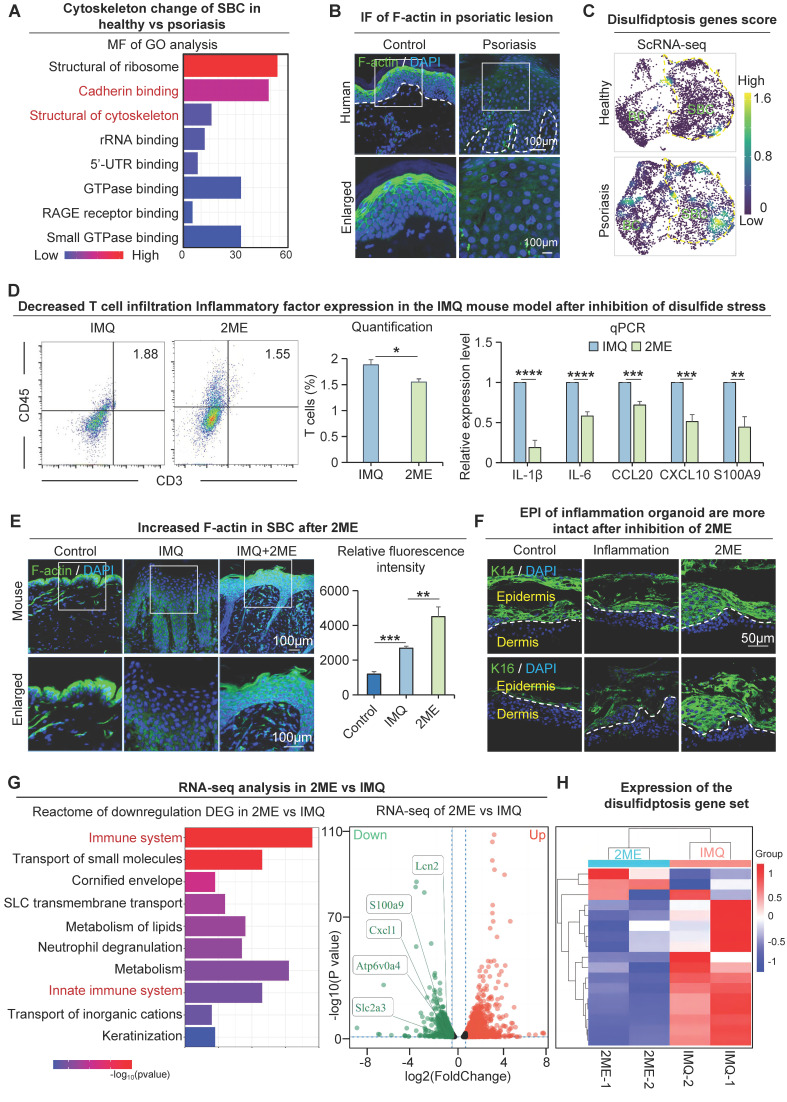
Disulfide stress mediates immature differentiation of upper basal cells in psoriasis. **A.** Molecular functional enrichment analysis of differential genes in upper basal cells from human healthy and psoriatic skin. **B.** Immunofluorescence of F-actin reveals cytoskeletal changes in cells from human healthy and psoriatic skin. **C.** Single-cell scoring of the disulfide death gene set shows active disulfide stress in upper basal cells in psoriasis. **D.** Flow cytometry analysis of T cell infiltration after inhibition of disulfide stress (left), and qRT-PCR showing expression levels of psoriasis-associated inflammatory factors after inhibition of disulfide stress (right). N=3, ****p < 0.0001, ***p < 0.001, **p < 0.01, *p < 0.05. **E.** Immunofluorescence staining of F-actin in control, IMQ, and IMQ+2ME groups shows cytoskeletal changes; Statistical analysis of fluorescence quantification of F-actin staining in control, IMQ, and IMQ+2ME groups. N=3, ***p < 0.001, **p < 0.01. Scale bar, 100 μm. **F.** Immunofluorescence staining of K14 and K16 in planar inflammatory skin organoids to show epidermal thickness changes after 2ME treatment. Scale bar, 50 μm. **G.** Bulk RNA-seq analysis of the downstream pathway of disulfide poisoning. **H.** Clustered heatmaps show reduced expression of the disulfidptosis gene set after 2ME.

Next, we investigated the molecular mechanisms that lead to changes in the suprabasal cells with low glucose metabolic activity in psoriasis. We conducted a GO analysis of differentially expressed genes in human suprabasal cells from the psoriasis group and control. The molecular functions revealed that pathways, including cadherin binding and structural constituent of the cytoskeleton, were significantly enriched in these groups (Figure 7A). To examine the epidermal cytoskeletal changes in psoriasis, we conducted immunofluorescence staining to label the cytoskeletal protein F-actin. The results indicate a reduction and disordered expression of F-actin in the human suprabasal cells of psoriasis compared to the control, psoriasis-like mouse models show the same results (Figure 7B, S7A). We used the barrier molecule KLK7 to show the location of upper basal cell expression in the skin (Figure S1B).

We next asked what causes the alterations in the suprabasal cytoskeleton in psoriatic skin. Our study suggests that the glucose content of suprabasal cells is reduced in psoriasis and that GLUT1-mediated uptake of glucose by basal cells may further reduce the glucose content of suprabasal cells (Figure 1J). We also observed increased expression of SLC7A11 in suprabasal cells in psoriatic skin (Figure 5E-F, S5B). This may lead to the accumulation of intracellular cystine, causing disulfide stress and ultimately resulting in cell death 41. To provide additional evidence that incomplete maturation of psoriatic suprabasal cells is caused by oxidative stress, we analyzed the disulfite death dataset 41 using scRNA-seq data from the human healthy and psoriatic cohorts, and found that the disulfite death scores for suprabasal cells were higher in the psoriatic group than the control (Figure 7C, S7B). We proposed that the incomplete differentiation of suprabasal cells might be caused by disulfide stress triggered by low glucose metabolism materials, resulting in cytoskeletal contraction and a subsequent change in the cellular state (Figure S7C). Since the conditions for disulfidptosis to occur intracellularly are high expression of SLC7A11 in cells and decreased expression of glucose and NADPH. The source of intracellular NADPH is the pentose phosphate pathway (PPP), and analysis of the spatial transcriptome results indicates that key genes of the PPP pathway are minimally expressed in the suprabasal cells of psoriatic tissues (Figure S7D). These results suggest that the supply of NADPH in psoriatic upper basal cells is insufficient to satisfy the intracellular process of reducing cystine to cysteine, resulting in disulfide stress and ultimately leading to aberrant cell differentiation or death.

By injecting a reducing agent that prevents disulfide stress (2ME) into the dorsal skin of the IMQ-induced psoriasis-like mice, we found that IMQ + 2ME-treated mice had milder psoriasis-like phenotype and a lower PASI score compared to the IMQ-treated group (Figure S7E). H&E staining revealed that the epidermis of IMQ + 2ME-treated mice was thinner, with fewer ridges extending downwards and lower baker's scores compared to the IMQ-treated group (Figure S7F). Flow cytometry analysis showed reduced T-cell infiltration in the IMQ + 2ME-treated group compared to the IMQ-treated group, and qPCR results showed reduced production and secretion of psoriasis-associated factors in the IMQ + 2ME-treated group compared to the IMQ-treated group (Figure 7D). Immunofluorescence staining of COL17A1 and P63 revealed a decrease of proliferating cells within the IMQ + 2ME-treated mice group (Figure S7G). Immunofluorescence staining and statistical analysis revealed higher and more organized levels of F-actin expression in the IMQ + 2ME-treated group in comparison with the healthy mouse group (Figure 7E). Epidermal thickness increased in the inflammatory organoids model after K14 and K16 treatments, suggesting a better epidermal cell state after 2ME treatment. (Figure 7F). Immunofluorescence staining and statistical analysis showed that F-actin expression was significantly increased and more ordered in suprabasal cells in the GLUT1-inhibited group compared with the IMQ-treated group (Figure S7H). To explore the molecular alterations after inhibition of disulfide stress, we performed transcriptome sequencing analyses on samples after 2ME treatment. We did a Reactome enrichment analysis of down-regulated genes after treatment using 2ME, and the results showed that the immune system and innate immune response pathways were significantly enriched. Moreover, the expression of disulfide death-related genes was significantly decreased after 2ME treatment (Figure 7G-H). The above results indicated that psoriasis disulfide stress and immune response were reduced after using a disulfide reducer, and finally, leading to alleviated symptoms of psoriasis. These results suggest that preventing disulfide stress can also alleviate psoriasis symptoms.

## Discussion

The main characteristics of psoriasis are hyperproliferation of basal cells, parakeratosis or hyperkeratosis of keratinocytes 42. It takes about 28 days for normal skin epidermal cells to shed from newborn to death, while the metabolic cycle of patients with psoriasis is greatly shortened to 4-5 days. Therefore, metabolic disorders exacerbate psoriasis progression. From a perspective of metabolic competition, this study is the careful mapping of the metabolism status of basal and suprabasal cells in normal and psoriatic skin and shows that the highly adaptive basal cells with high energy demand take up glucose from surrounding suprabasal cells, leading to the hyperproliferation and thickening of the epidermis in psoriatic skin. Moreover, the suprabasal cells express a high level of SLC7A11, resulting in cytoskeleton shrinkage and immature differentiation. Together, these cause psoriasis progression.

### Metabolic competition maintains hyperproliferation of epidermal cells in psoriasis

In psoriasis, it has been shown that external antigen triggers innate immunity and activates plasmacytoid dendritic cells, which produce IL-23 and TNF-alpha to differentiate naive T cells into Th17 cells. Then Th17 cells produce IL-17 to promote epidermal proliferation 1-3. The basal cell responds to IL-17 stimuli for proliferation and differentiation to produce a thickened epidermis. Here, the inflammatory cytokines may tilt the balance toward the psoriatic basal cell states. Metabolic competition and symbiosis occur due to the continuous interaction between cancer cells and stromal cells during highly proliferative tumourigenesis 25. Cellular competition mechanisms have also been used to explain the heterogeneity of immune cells in tumors, and there is also nutrient competition between tumor cells and immune cells, where tumors inhibit the function of tumor-infiltrating T-cells by competitive uptake of glucose 43, where the dominant tumor clones with unique metabolic profiles (possibly glycolysis) escape killing by T-cells, and tumor cells without such metabolic adaptation tumor cells become loser clones and are eliminated by T cells 43. The current theory is that dominant clones with higher glycolytic activity express lower levels of IRF1 and CXCL10 immunostimulatory molecules, thereby allowing them to avoid recognition by T cells. A possible explanation for the phenomenon of skin hyperproliferation observed in psoriasis is then a loss of control of cell competition mechanisms triggered by metabolic reprogramming. The "obese cell hypothesis" (i.e., cells that have gained a competitive advantage through metabolic alterations can survive and reproduce in cell populations) has been widely described in tumor biology studies 44. In the context of psoriasis, this phenomenon may also be applicable, suggesting that basal cells in diseased skin win the competitive environment due to metabolic advantages, leading to rapid renewal and increased thickness of the skin surface.

The present study showed that psoriasis has a significantly higher expression of GLUT1 in basal cells compared to the suprabasal cells. GLUT1 translocates a significant amount of glucose to basal cells, wherein glucose acts as a crucial substrate for protein biosynthesis, implying a higher protein synthesis rate in these cells. In Drosophila, cells that overexpress MYC demonstrate greater adaptability than wild-type cells. These MYC-overexpressing cells prompt the elimination of wild-type cells in mixed-population organisms, establishing them as “super-competitors” 45. One could speculate that this can promote the adaptation of the basal cells towards becoming "super-competitors" of glucose.

Additionally, cellular competitive behavior correlates with energy metabolism. In the Madin-Darby canine kidney cell culture, the peripheral wild-type cells were eliminated to remove the oncogenic mutant form of RasG12V. Cells expressing RasG12V show reduced mitochondrial oxidative phosphorylation, which is hindered by pyruvate dehydrogenase kinase 4 (PDK4). Reducing PDK4 in RasG12V cells slowed down their elimination, indicating a potential role of oxidative phosphorylation in controlling cellular competition 46. The present study revealed that metabolically adapted basal cells regulate their highly proliferative behavior through the sugar-oxidative phosphorylation axis. These findings suggest that cellular competition mechanisms are not only relevant to the developmental homeostasis of normal organs but also to the cellular plasticity and pathological homeostasis of the cellular environment. It also provides new ideas to explain the progression of psoriasis from the perspective of cellular competition.

### Disulfide stress-mediated disulfide death is involved in the immature differentiation of suprabasal cells in psoriasis

The suprabasal cells within psoriatic skin acting as the loser cells for metabolic competition may lack adequate nutrients, resulting in premature differentiation and the formation of scaly and white patches on the skin surface. Our investigation revealed that the suprabasal cells in psoriasis show an altered cellular state characterized by reduced and disorganized F-actin expression and cytoskeletal contraction. What are the molecular mechanisms responsible for the changes observed in the suprabasal cells? To begin with, suprabasal cells that fail in metabolic competition, exhibit a reduction in glucose levels, and reduced expression of key genes of the PPP. This results in a decrease in the levels of NADPH within the cells. We observed a higher expression of SLC7A11 in suprabasal cells than in basal cells of psoriatic skin. This facilitates the entry of disulfides (such as cystine) into the suprabasal cells. In cells, NADPH reduces disulfides during crucial moments to ensure the adequate survival of the cells. It has been shown that glucose-deficient cancer cells expressing high levels of SLC7A11 accumulate large amounts of disulphide molecules. This leads to abnormal disulphide bonding between the actin cytoskeletal proteins, resulting in the collapse of the actin network and cell death 41. Our study also revealed abnormalities in the suprabasal cell actin cytoskeleton in psoriasis. The addition of disulphide inhibitor prevented disulfide stress and successfully restored the cytoskeletal abnormalities and the psoriasis phenotype. It is likely that this cell death co-mediates the formation of cytopathic states in psoriasis.

### The subtle balance of redox maintains the progression of psoriasis

In living organisms, superoxide anion, hydrogen peroxide produced by mitochondrial respiration, is the main source of ROS 47. The present study also confirmed that basal cells in psoriasis show enhanced oxidative phosphorylation activity, indicating that mitochondria are in a highly active respiratory state. In the body, a delicate balance between normal ROS production and the cellular antioxidant defense system allows the body to function properly 48. Once this balance is disrupted, the harmful accumulation of ROS will put the body in a state of oxidative stress 49. This in turn leads to a variety of adverse effects, including cell and tissue damage 50. On the other hand, ROS is gradually being shown to play a dual role in certain diseases, even though too much of it can cause multiple damages to the body. It is considered to have broad therapeutic potential in anti-cancer and regenerative medicine 51.

In psoriasis models, aberrant accumulation of ROS increases antioxidant depletion *in vivo*, leading to an imbalance in the body's oxidative defense system and further exacerbating the pathological progression of psoriasis. In the context of the present study, the observed elevation of glutathione levels may be a natural response to ROS accumulation by activating glutathione metabolic pathways to counteract the increase in ROS *in vivo*. The ability to slow down the progression of psoriasis, as demonstrated by glutathione supplementation in this study, suggests an important role for antioxidants in maintaining redox balance and preventing the onset of oxidative stress. Furthermore, it has also been found that high levels of ROS can prevent imiquimod-induced psoriasis-like symptoms by promoting the function of regulatory T cells (Treg) 52. Thus, a delicate balance of redox should be maintained in psoriasis to sustain normal disease progression. Such findings may provide new perspectives for psoriasis-rated therapy, especially in the application of antioxidant strategies.

In conclusion, our study uncovered that cellular competition triggers a metabolic-molecular cascade response, promoting the pathological hyperproliferation of epidermal cells in psoriasis. This study innovated the understanding of the significance of cell competition to the metabolic regulation of tissues and organs during disease progression. Furthermore, this study presents a novel mode of cell death in psoriasis, referred to as disulfide death in regulating suprabasal cell differentiation in psoriasis. This offers new avenues for further research and even psoriasis treatment.

## Supplementary Material

Supplementary figures and tables, materials and methods.

## Abbreviations

KEGG: kyoto encyclopedia of genes and genomes
OXPHOS: oxidative phosphorylation
PASI: psoriasis lesion area and severity index
DEG: differentially expressed genes
ST: spatial transcriptome
PFA: paraformaldehyde
PI: propidium iodide
H&E: hematoxylin and eosin
scRNA-seq: single-cell RNA sequencing
UMAP: uniform manifold approximation and projection
IMQ: imiquimod
BC: basal cell
SBC: suprabasal cell
FBI: fibroblasts I
FBII: fibroblasts II
GO: gene ontology
ROT: Rotenone
3D: 3-dimensional
D0: day 1
D3: day 3
D4: day 4
D7: day 7
GSH: glutathione
WB: western blot
BSO: buthionine sulfoximine
2ME: 2-Methoxyestradiol

## References

[1] Karami H, Khalilzadeh Arjmandi H, Salehifar E, Darvishi-Khezri H, Dabirian M, Kosaryan M (2021). A double-blind, controlled, crossover trial of amlodipine on iron overload status in transfusion dependent β-thalassemia patients. Int J Clin Pract.

[2] Christophers E (2001). Psoriasis-epidemiology and clinical spectrum. Clin Exp Dermatol.

[3] Ding X, Wang T, Shen Y, Wang X, Zhou C, Tian S (2012). Prevalence of psoriasis in China: a population-based study in six cities. Eur J Dermatol.

[4] Gladman DD, Antoni C, Mease P, Clegg DO, Nash P (2005). Psoriatic arthritis: epidemiology, clinical features, course, and outcome. Ann Rheum Dis.

[5] Pasparakis M, Haase I, Nestle FO (2014). Mechanisms regulating skin immunity and inflammation. Nat Rev Immunol.

[6] Łuczaj W, Gęgotek A, Skrzydlewska E (2021). Analytical approaches to assess metabolic changes in psoriasis. J Pharm Biomed Anal.

[7] Lian N, Shi LQ, Hao ZM, Chen M (2020). Research progress and perspective in metabolism and metabolomics of psoriasis. Chin Med J (Engl).

[8] Kamleh MA, Snowden SG, Grapov D, Blackburn GJ, Watson DG, Xu N (2015). LC-MS metabolomics of psoriasis patients reveals disease severity-dependent increases in circulating amino acids that are ameliorated by anti-TNFα treatment. J Proteome Res.

[9] Kang H, Li X, Zhou Q, Quan C, Xue F, Zheng J (2017). Exploration of candidate biomarkers for human psoriasis based on gas chromatography-mass spectrometry serum metabolomics. Br J Dermatol.

[10] Wroński A, Wójcik P (2022). Impact of ROS-Dependent Lipid Metabolism on Psoriasis Pathophysiology. Int J Mol Sci.

[11] Herbert D, Franz S, Popkova Y, Anderegg U, Schiller J, Schwede K (2018). High-Fat Diet Exacerbates Early Psoriatic Skin Inflammation Independent of Obesity: Saturated Fatty Acids as Key Players. J Invest Dermatol.

[12] Lou F, Sun Y, Xu Z, Niu L, Wang Z, Deng S (2020). Excessive Polyamine Generation in Keratinocytes Promotes Self-RNA Sensing by Dendritic Cells in Psoriasis. Immunity.

[13] Zhang Z, Zi Z, Lee EE, Zhao J, Contreras DC, South AP (2018). Differential glucose requirement in skin homeostasis and injury identifies a therapeutic target for psoriasis. Nat Med.

[14] Vander Heiden MG, Cantley LC, Thompson CB (2009). Understanding the Warburg effect: the metabolic requirements of cell proliferation. Science.

[15] Michalek RD, Gerriets VA, Jacobs SR, Macintyre AN, MacIver NJ, Mason EF (2011). Cutting edge: distinct glycolytic and lipid oxidative metabolic programs are essential for effector and regulatory CD4+ T cell subsets. J Immunol.

[16] Chen C, Yi X, Liu P, Li J, Yan B, Zhang D (2023). CD147 Facilitates the Pathogenesis of Psoriasis through Glycolysis and H3K9me3 Modification in Keratinocytes. Research (Wash D C).

[17] Bondar T, Medzhitov R (2010). p53-mediated hematopoietic stem and progenitor cell competition. Cell Stem Cell.

[18] Snippert HJ, van der Flier LG, Sato T, van Es JH, van den Born M, Kroon-Veenboer C (2010). Intestinal crypt homeostasis results from neutral competition between symmetrically dividing Lgr5 stem cells. Cell.

[19] Prillaman M (2022). Alzheimer's drug slows mental decline in trial - but is it a breakthrough?. Nature.

[20] Kozar S, Morrissey E, Nicholson AM, van der Heijden M, Zecchini HI, Kemp R (2013). Continuous clonal labeling reveals small numbers of functional stem cells in intestinal crypts and adenomas. Cell Stem Cell.

[21] Corominas-Murtra B, Scheele C, Kishi K, Ellenbroek SIJ, Simons BD, van Rheenen J (2020). Stem cell lineage survival as a noisy competition for niche access. Proc Natl Acad Sci U S A.

[22] Ellis SJ, Gomez NC, Levorse J, Mertz AF, Ge Y, Fuchs E (2019). Distinct modes of cell competition shape mammalian tissue morphogenesis. Nature.

[23] Liu N, Matsumura H, Kato T, Ichinose S, Takada A, Namiki T (2019). Stem cell competition orchestrates skin homeostasis and ageing. Nature.

[24] Lei M, Chuong CM (2018). Epidermal Darwinism and Competitive Equilibrium within the Epidermis. Cell Stem Cell.

[25] Dias AS, Almeida CR, Helguero LA, Duarte IF (2019). Metabolic crosstalk in the breast cancer microenvironment. Eur J Cancer.

[26] Zhao Q, Yu J, Zhou H, Wang X, Zhang C, Hu J (2023). Intestinal dysbiosis exacerbates the pathogenesis of psoriasis-like phenotype through changes in fatty acid metabolism. Signal Transduct Target Ther.

[27] Lei M, Schumacher LJ, Lai YC, Juan WT, Yeh CY, Wu P (2017). Self-organization process in newborn skin organoid formation inspires strategy to restore hair regeneration of adult cells. Proc Natl Acad Sci U S A.

[28] Lei M, Harn HI, Li Q, Jiang J, Wu W, Zhou W (2023). The mechano-chemical circuit drives skin organoid self-organization. Proc Natl Acad Sci U S A.

[29] Lei M, Jiang J, Wang M, Wu W, Zhang J, Liu W (2023). Epidermal-dermal coupled spheroids are important for tissue pattern regeneration in reconstituted skin explant cultures. NPJ Regen Med.

[30] Wang M, Zhou X, Zhou S, Wang M, Jiang J, Wu W (2023). Mechanical force drives the initial mesenchymal-epithelial interaction during skin organoid development. Theranostics.

[31] Meng Y, Wang M, Xie X, Di T, Zhao J, Lin Y (2017). Paeonol ameliorates imiquimod-induced psoriasis-like skin lesions in BALB/c mice by inhibiting the maturation and activation of dendritic cells. Int J Mol Med.

[32] Wang Y, Han D, Huang Y, Dai Y, Wang Y, Liu M (2023). Oral administration of punicalagin attenuates imiquimod-induced psoriasis by reducing ROS generation and inflammation via MAPK/ERK and NF-κB signaling pathways. Phytother Res.

[33] Gao Y, Yao X, Zhai Y, Li L, Li H, Sun X (2021). Single cell transcriptional zonation of human psoriasis skin identifies an alternative immunoregulatory axis conducted by skin resident cells. Cell Death Dis.

[34] Yan R, Zhang H, Ma Y, Lin R, Zhou B, Zhang T (2022). Discovery of Muscle-Tendon Progenitor Subpopulation in Human Myotendinous Junction at Single-Cell Resolution. Research (Wash D C).

[35] Liu Q, Long Q, Zhao J, Wu W, Lin Z, Sun W (2023). Cold-Induced Reprogramming of Subcutaneous White Adipose Tissue Assessed by Single-Cell and Single-Nucleus RNA Sequencing. Research (Wash D C).

[36] Ma F, Plazyo O, Billi AC, Tsoi LC, Xing X, Wasikowski R (2023). Single cell and spatial sequencing define processes by which keratinocytes and fibroblasts amplify inflammatory responses in psoriasis. Nat Commun.

[37] Zong J, Cheng J, Fu Y, Song J, Pan W, Yang L (2020). Serum Metabolomic Profiling Reveals the Amelioration Effect of Methotrexate on Imiquimod-Induced Psoriasis in Mouse. Front Pharmacol.

[38] Cibrian D, de la Fuente H, Sánchez-Madrid F (2020). Metabolic Pathways That Control Skin Homeostasis and Inflammation. Trends Mol Med.

[39] Zhang M, Wang M, Jiang J, Liu W, Zhou S, Wang D (2023). COX2-ATP Synthase Regulates Spine Follicle Size in Hedgehogs. Int J Biol Sci.

[40] Hu J, Bian Q, Ma X, Xu Y, Gao J (2022). A double-edged sword: ROS related therapies in the treatment of psoriasis. Asian J Pharm Sci.

[41] Liu X, Nie L, Zhang Y, Yan Y, Wang C, Colic M (2023). Actin cytoskeleton vulnerability to disulfide stress mediates disulfidptosis. Nat Cell Biol.

[42] Qiu J, Villa M, Sanin DE, Buck MD, O'Sullivan D, Ching R (2019). Acetate Promotes T Cell Effector Function during Glucose Restriction. Cell Rep.

[43] Rabinovich RV, Lee SJ (2018). Proximal Row Carpectomy Using Decellularized Dermal Allograft. J Hand Surg Am.

[44] Parker TM, Henriques V, Beltran A, Nakshatri H, Gogna R (2020). Cell competition and tumor heterogeneity. Semin Cancer Biol.

[45] de la Cova C, Abril M, Bellosta P, Gallant P, Johnston LA (2004). Drosophila myc regulates organ size by inducing cell competition. Cell.

[46] Kon S, Ishibashi K, Katoh H, Kitamoto S, Shirai T, Tanaka S (2017). Cell competition with normal epithelial cells promotes apical extrusion of transformed cells through metabolic changes. Nat Cell Biol.

[47] Sies H, Jones DP (2020). Reactive oxygen species (ROS) as pleiotropic physiological signalling agents. Nat Rev Mol Cell Biol.

[48] Go YM, Jones DP (2017). Redox theory of aging: implications for health and disease. Clin Sci (Lond).

[49] Sies H (2017). Hydrogen peroxide as a central redox signaling molecule in physiological oxidative stress: Oxidative eustress. Redox Biol.

[50] Tan DQ, Suda T (2018). Reactive Oxygen Species and Mitochondrial Homeostasis as Regulators of Stem Cell Fate and Function. Antioxid Redox Signal.

[51] Perillo B, Di Donato M, Pezone A, Di Zazzo E, Giovannelli P, Galasso G (2020). ROS in cancer therapy: the bright side of the moon. Exp Mol Med.

[52] Kim HR, Lee A, Choi EJ, Hong MP, Kie JH, Lim W (2014). Reactive oxygen species prevent imiquimod-induced psoriatic dermatitis through enhancing regulatory T cell function. PLoS One.

