# Supplementary material for: Energy competition remodels the metabolic glucose landscape of psoriatic epidermal cells
Source: Theranostics. 2024 May 27;14(8):3339–57. doi: 10.7150/thno.93764 (PMC11155411; doi:10.7150/thno.93764)
Supplement: Supplementary file 1 — Supplementary figures and tables, materials and methods. [file thnov14p3339s1.pdf]

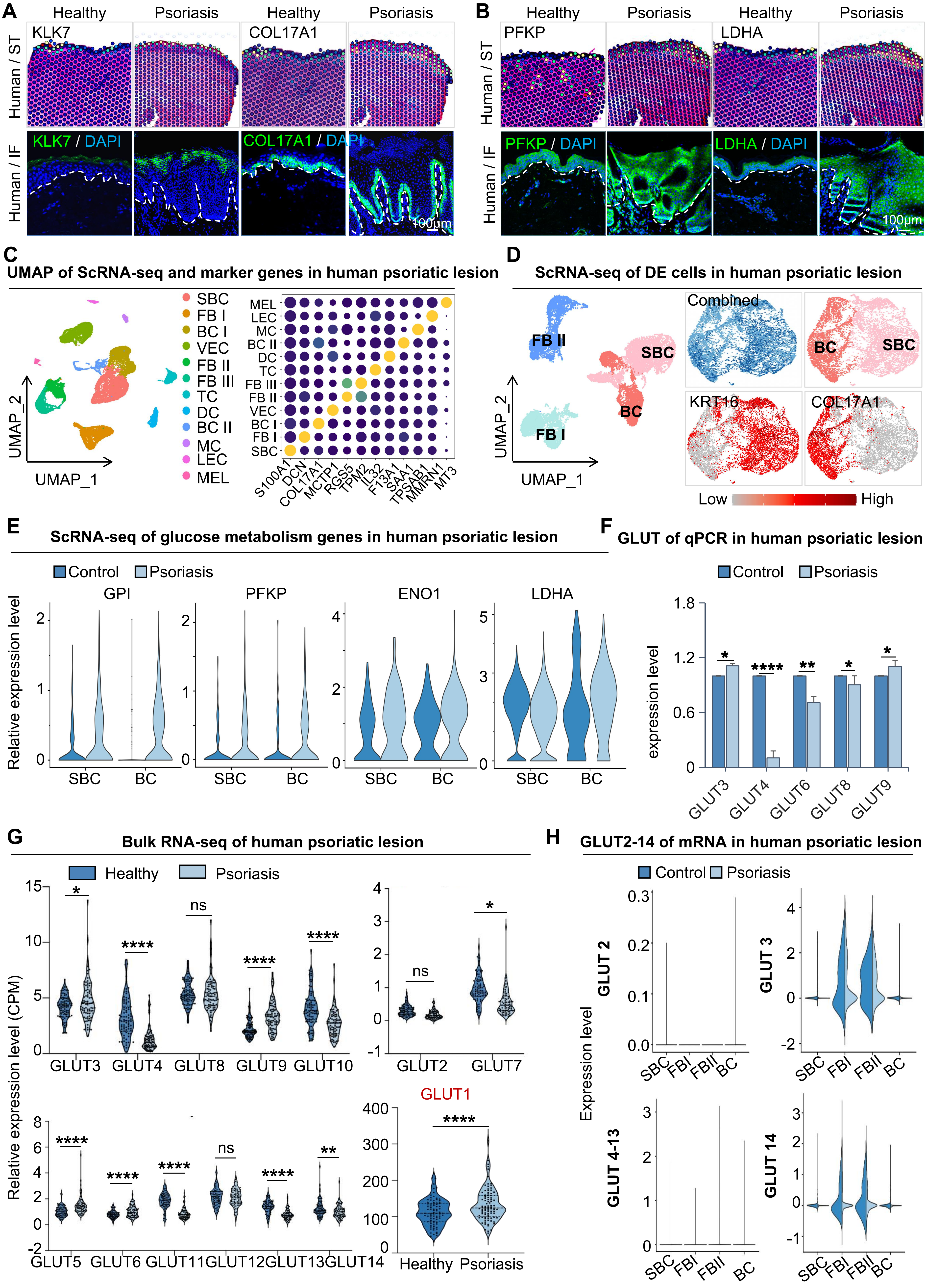
A
Healthy
Psoriasis
Healthy
Psoriasis
Human / ST
Human / IF
KLK7
COL17A1
KLK7 / DAPI
COL17A1 / DAPI
100μm
B
PFKP
LDHA
PFKP / DAPI
LDHA / DAPI
C
UMAP of ScRNA-seq and marker genes in human psoriatic lesion
SBC
FB I
BC I
VEC
FB II
FB III
TC
DC
BC II
MC
LEC
MEL
UMAP_1
UMAP_2
S100A1
DCN
COL17A1
MCTP1
RGS5
TPM2
IL32
F13A1
SAA1
TPSAB1
MMRN1
MT3
D
ScRNA-seq of DE cells in human psoriatic lesion
Combined
BC
KRT16
Low
High
E
ScRNA-seq of glucose metabolism genes in human psoriatic lesion
Control
Psoriasis
GPI
PFKP
ENO1
Relative expression level
F
GLUT of qPCR in human psoriatic lesion
expression level
GLUT3
GLUT4
GLUT6
GLUT8
GLUT9
G
Bulk RNA-seq of human psoriatic lesion
Healthy
Relative expression level (CPM)
GLUT10
GLUT2
GLUT7
GLUT1
GLUT5
GLUT11
GLUT12
GLUT13
GLUT14
ns
H
GLUT2-14 of mRNA in human psoriatic lesion
GLUT 2
GLUT 3
GLUT 4-13
GLUT 14
Expression level
FBI
FBII

Figure S1. Multiomic analysis shows active basal cell layer glucose metabolism in psoriasis.

A. Spatial transcriptome and immunofluorescence staining shows the location of expression of basal cell marker COL17A1 and upper basal cell marker KLK7 in human healthy and psoriatic skin. Scale bar, 100 μm.

B. Spatial transcriptome and immunofluorescence staining of gluconeogenic genes show PFKP and LDHA expression in the basal cell layer in human psoriatic skin. Scale bar, 100 μm.

C. Unbiased clustering method for UMAP plots of human healthy and psoriatic skin data and marker genes map of different cell subpopulations in single-cell data.

D. UMAP of dermal and epidermal cells by unbiased clustering method (left); KRT16 and COL17A1 show the location of basal and suprabasal cell clusters.

E. Expression analysis of glycometabolic genes GPI, PFKP, ENO1, and LDHA by scRNA-seq in human health and psoriatic skin.

F. qRT-PCR shows expression of the GLUT family of glucose transporter proteins in human psoriatic skin. N=3, ****$p < 0.0001$, **$p < 0.01$, *$p < 0.05$.

G. Bulk RNA-seq data of human health and psoriatic skin shows expression levels of GLUT1-14. N=82, ****$p < 0.0001$, **$p < 0.01$, *$p < 0.05$ ns, no significant change.

H. Single-cell sequencing data reveal the location of expression of the GLUT family of glucose transporter proteins in human psoriatic skin.

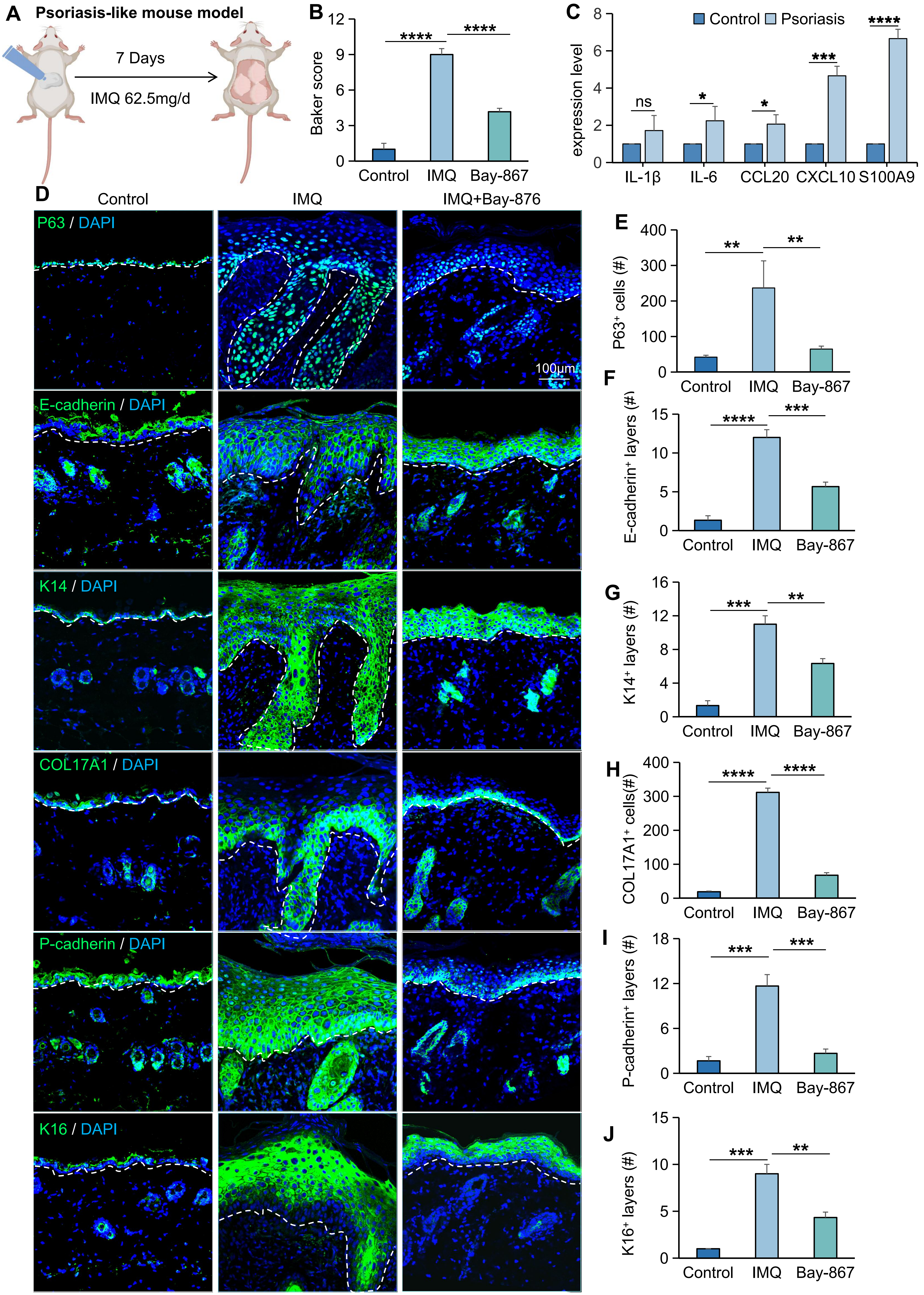
A
Psoriasis-like mouse model
7 Days
IMQ 62.5mg/d
B
Baker score
Control
IMQ
Bay-867
C
Control
Psoriasis
expression level
IL-1β
IL-6
CCL20
CXCL10
S100A9
D
Control
IMQ
IMQ+Bay-876
P63 / DAPI
E-cadherin / DAPI
K14 / DAPI
COL17A1 / DAPI
P-cadherin / DAPI
K16 / DAPI
100μm
E
P63+ cells (#)
F
E-cadherin+ layers (#)
G
K14+ layers (#)
H
COL17A1+ cells(#)
I
P-cadherin+ layers (#)
J
K16+ layers (#)

Figure S2. Thinning of the epidermis of IMQ model mice after Bay-867 treatment.

A. Schematic of imiquimod-induced psoriasis-like mouse model.

B. Baker scores of dorsal H&E staining of mice in control, IMQ, and IMQ+Bay-867 groups. N=3, ****$p < 0.0001$.

C. qRT-PCR to analyze the expression levels of psoriasis-related inflammatory factors in the IMQ group. N=3, ****$p < 0.0001$, ***$p < 0.001$, *$p < 0.05$ ns, no significant change.

D. Immunofluorescence staining of P63, E-cadherin, K14, COL17A1, P-cadherin, and K16 shows changes in epidermal thickness in psoriasis-like mice after Bay-867 treatment. Scale bar, 100 μm.

E. Statistical analysis of $P63^+$ cells in control, IMQ, and IMQ+Bay-867 groups. N=3, **$p < 0.01$.

F. Statistical analysis of E-cadherin$^+$ layers in control, IMQ, and IMQ+Bay-867 groups. N=3, ****$p < 0.0001$, ***$p < 0.001$.

G. Statistical analysis of $K14^+$ layers in the control, IMQ, and IMQ+Bay-867 groups. N=3, ***$p < 0.001$, **$p < 0.01$.

H. Statistical analysis of $COL17A1^+$ cells in control, IMQ, and IMQ+Bay-867 groups. N=3, ****$p < 0.0001$.

I. Statistical analysis of P-cadherin$^+$ layers in control, IMQ, and IMQ+Bay-867 groups. N=3, ***$p < 0.001$.

J. Statistical analysis of $K16^+$ layers in control, IMQ, and IMQ+Bay-867 groups. N=3, ***$p < 0.001$, **$p < 0.01$.

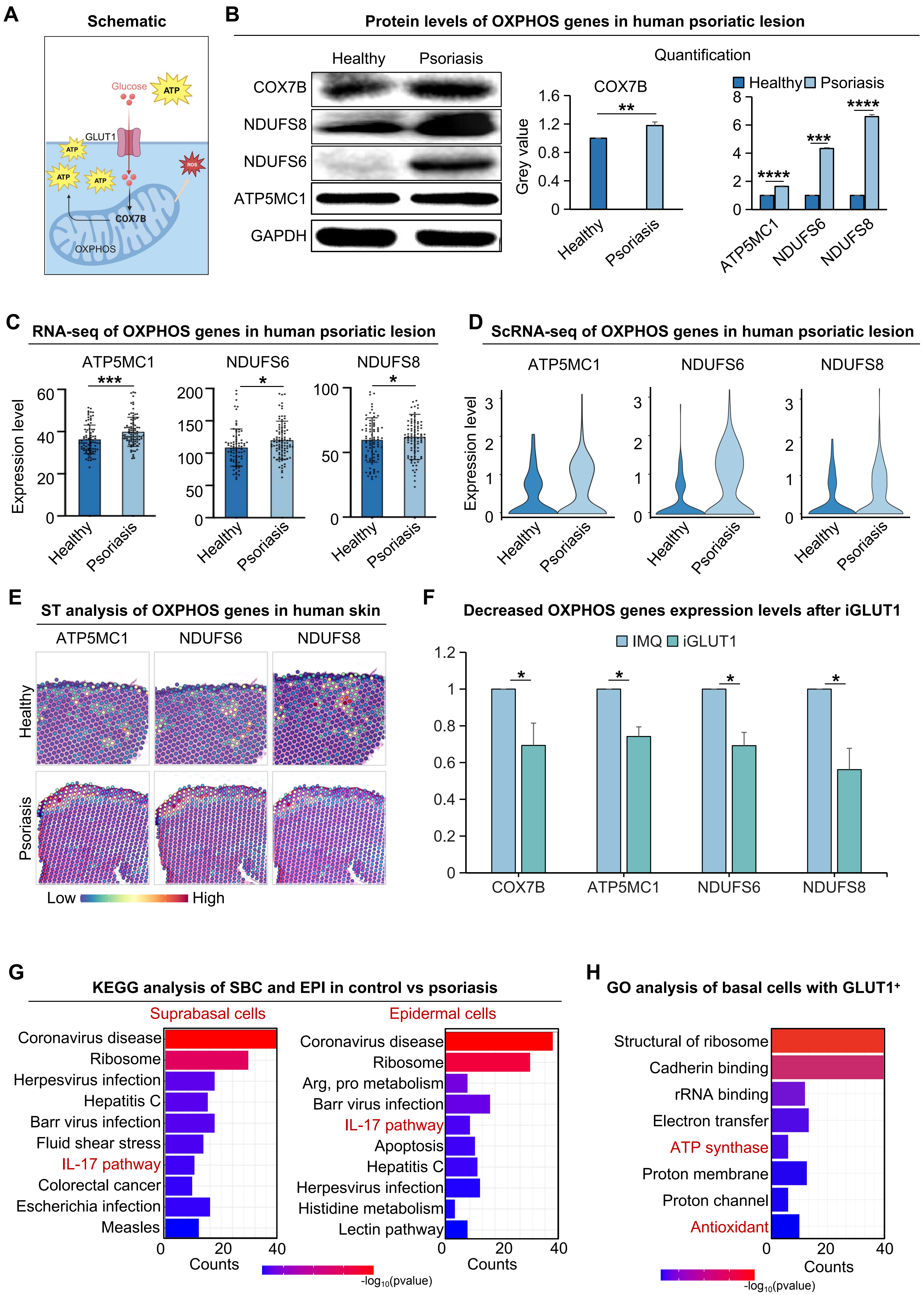
A
Schematic
Glucose
ATP
GLUT1
ROS
COX7B
OXPHOS
B
Protein levels of OXPHOS genes in human psoriatic lesion
Healthy
Psoriasis
COX7B
NDUFS8
NDUFS6
ATP5MC1
GAPDH
Quantification
Grey value
Healthy Psoriasis
ATP5MC1
NDUFS6
NDUFS8
C
RNA-seq of OXPHOS genes in human psoriatic lesion
Expression level
D
ScRNA-seq of OXPHOS genes in human psoriatic lesion
E
ST analysis of OXPHOS genes in human skin
Low
High
F
Decreased OXPHOS genes expression levels after iGLUT1
IMQ
iGLUT1
G
KEGG analysis of SBC and EPI in control vs psoriasis
Suprabasal cells
Epidermal cells
Coronavirus disease
Ribosome
Herpesvirus infection
Hepatitis C
Barr virus infection
Fluid shear stress
IL-17 pathway
Colorectal cancer
Escherichia infection
Measles
Arg, pro metabolism
Apoptosis
Histidine metabolism
Lectin pathway
Counts
-log10(pvalue)
H
GO analysis of basal cells with GLUT1+
Structural of ribosome
Cadherin binding
rRNA binding
Electron transfer
ATP synthase
Proton membrane
Proton channel
Antioxidant

Figure S3. Glucose metabolism promotes COX7B-mediated upregulation of OXPHOS in psoriatic basal cells.

A. Schematic of glucose-promoted OXPHOS upregulation.

B. Western blot shows protein expression levels of the OXPHOS gene in the human psoriatic lesions. N=3, ****$p < 0.0001$, ***$p < 0.001$, **$p < 0.01$.

C. qRT-PCR shows the gene expression levels of ATP5MC1, NDUFS6, and NDUFS8 in human psoriatic lesions. N=3, ***$p < 0.001$, *$p < 0.05$.

D. ScRNA-seq shows gene expression levels of ATP5MC1, NDUFS6, and NDUFS8 in human psoriatic lesions. N=3, ***$p < 0.001$, *$p < 0.05$.

E. Spatial transcriptome shows the expression location of ATP5MC1, NDUFS6, and NDUFS8 in human psoriatic lesions.

F. Decreased expression levels of OXPHOS-related genes after iGLUT1. N=3, *$p < 0.05$.

G. Differential genes KEGG enrichment analysis of suprabasal cells in human healthy and psoriatic skin (left); Differential genes KEGG enrichment analysis of epidermal cells in human healthy and psoriatic skin (right).

H. GO analysis of GLUT1+ basal cell differential genes in human healthy and psoriatic skin.

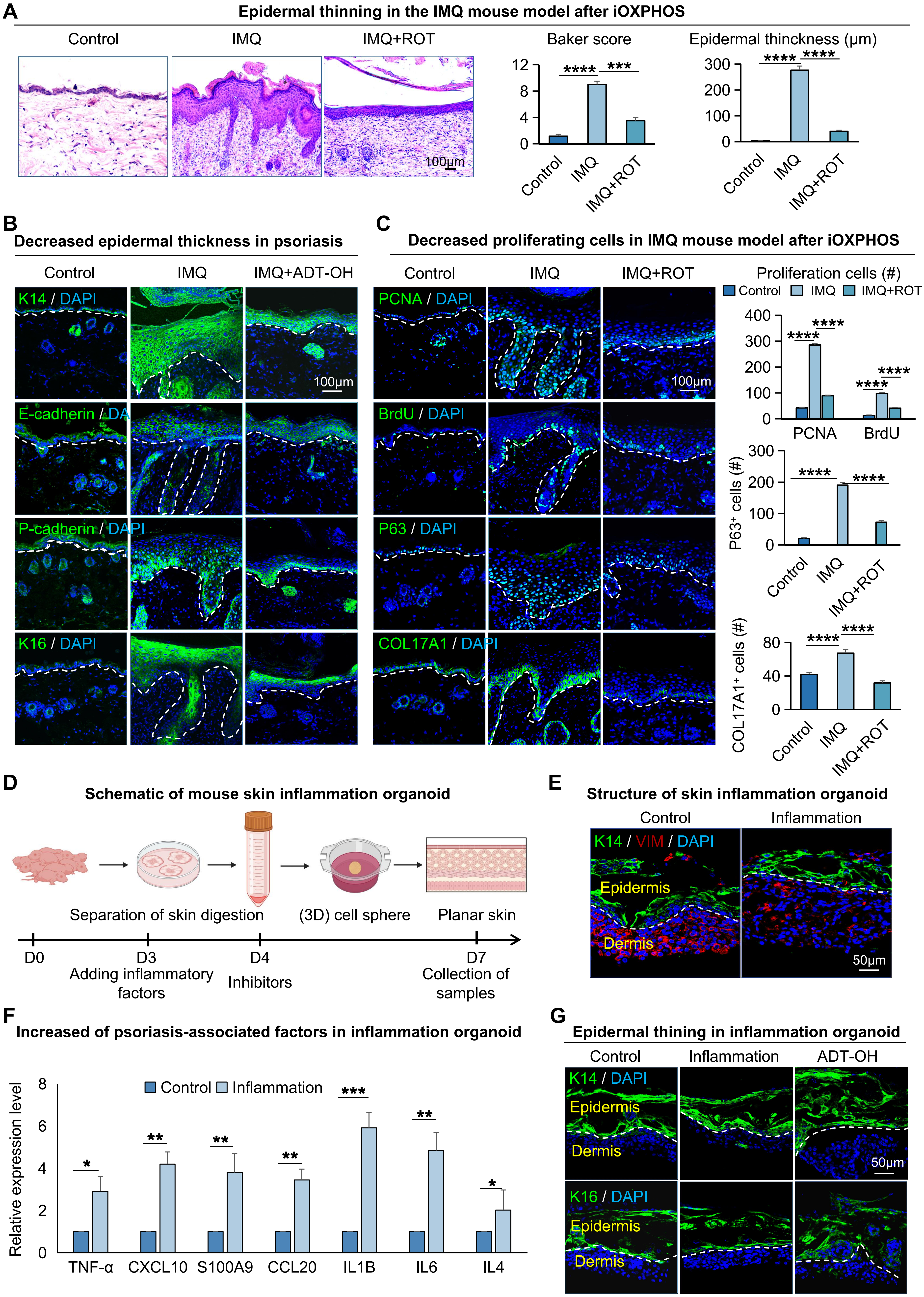

A
Epidermal thinning in the IMQ mouse model after iOXPHOS
Control
IMQ
IMQ+ROT
100μm
Baker score
Epidermal thinckness (μm)
B
Decreased epidermal thickness in psoriasis
Control
IMQ
IMQ+ADT-OH
K14 / DAPI
E-cadherin / DAPI
P-cadherin / DAPI
K16 / DAPI
C
Decreased proliferating cells in IMQ mouse model after iOXPHOS
PCNA / DAPI
BrdU / DAPI
P63 / DAPI
COL17A1 / DAPI
Proliferation cells (#)
P63+ cells (#)
COL17A1+ cells (#)
D
Schematic of mouse skin inflammation organoid
Separation of skin digestion
(3D) cell sphere
Planar skin
D0
D3
Adding inflammatory factors
D4
Inhibitors
D7
Collection of samples
E
Structure of skin inflammation organoid
Control
Inflammation
K14 / VIM / DAPI
Epidermis
Dermis
50μm
F
Increased of psoriasis-associated factors in inflammation organoid
Relative expression level
Control
Inflammation
TNF-α
CXCL10
S100A9
CCL20
IL1B
IL6
IL4
G
Epidermal thining in inflammation organoid
Control
Inflammation
ADT-OH
K14 / DAPI
K16 / DAPI
Epidermis
Dermis
50μm

Figure S4. Attenuated proliferative capacity of epidermal cells in IMQ-induced psoriasis-like mice after treatment with Rotenone and ADT-OH.

A. Representative H&E staining and baker score of the back of mice in the control, IMQ, and IMQ+ROT groups; Statistical analysis of epidermal thickness. Scale bar, 100 µm. N=3, ****$p < 0.0001$, ***$p < 0.001$.

B. Immunofluorescence staining of K14, E-cadherin, P-cadherin, and K16 shows skin thickness. Scale bar, 100 µm.

C. Immunofluorescence staining of PCNA, BrdU, P63, and Col17a1 shows diminished proliferative capacity of epidermal cells in the IMQ mouse model after ROT treatment. Statistical analysis of $PCNA^+$ cells , $BrdU^+$ cells, $P63^+$ cells and $Col17a1^+$ cells in control, IMQ, and IMQ+ROT groups. N=3, ****$p<0.0001$. Scale bar, 100 µm.

D. Schematic of inflammatory skin organoid.

E. Immunofluorescence staining of K14 and Vimentin shows the basic structure of inflammatory organoids. Scale bar, 50 µm.

F. qRT-PCR to analyze the expression levels of psoriasis-related inflammatory factors in inflammatory skin organoids. N=3, ***$p < 0.001$, **$p < 0.01$, *$p < 0.05$, ns, no significant change.

G. Immunofluorescence staining of K14 and K16 shows the epidermal thickness of inflammatory skin organoids. Scale bar, 50 µm.

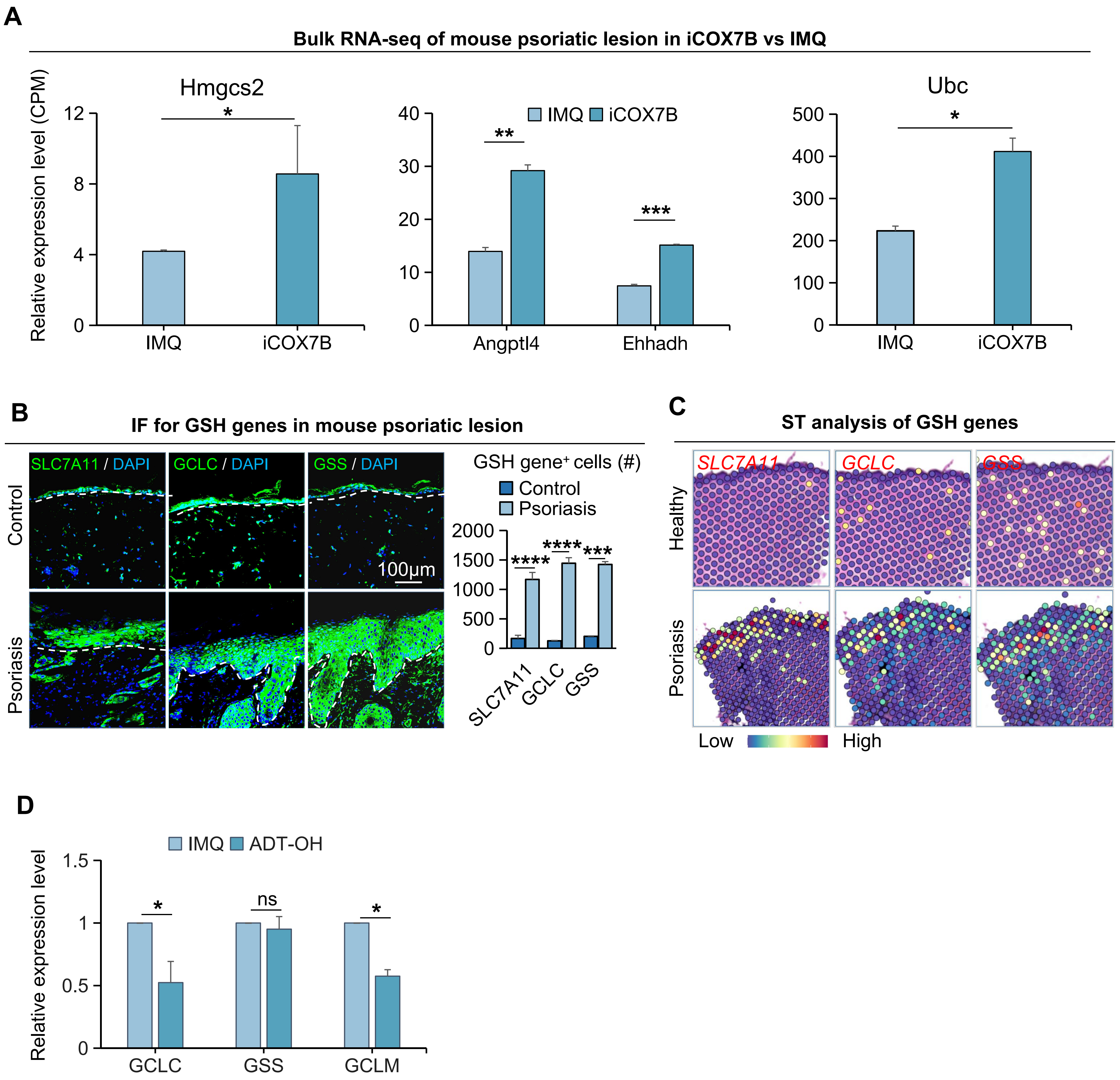
A
Bulk RNA-seq of mouse psoriatic lesion in iCOX7B vs IMQ
Hmgcs2
Relative expression level (CPM)
IMQ
iCOX7B
Ubc
Angptl4
Ehhadh
B
IF for GSH genes in mouse psoriatic lesion
SLC7A11 / DAPI
GCLC / DAPI
GSS / DAPI
Control
Psoriasis
100μm
GSH gene⁺ cells (#)
C
ST analysis of GSH genes
SLC7A11
GCLC
GSS
Healthy
Psoriasis
Low
High
D
IMQ
ADT-OH
Relative expression level
GCLC
GSS
GCLM
ns

Figure S5. Glutathione metabolism is significantly up-regulated in basal cells of psoriasis.

A. Bulk RNA-seq showed reduced expression of PPAR pathway-related genes after iCOX7B. N=3, ***$p < 0.001$, **$p < 0.01$, *$p < 0.05$.

B. Immunofluorescence staining of SLC7A11, GCLC, and GSS shows the location of their expression in mouse psoriatic lesions; GSH+ cells were statistically analyzed. Scale bar, 100 μm. N=3, ****$p < 0.0001$, ***$p < 0.001$.

C. Spatial transcriptome analysis shows the expression location of glutathione genes SLC7A11, GCLC, and GSS in human psoriatic lesions.

D. qRT-PCR showed reduced expression of glutathione synthesis-related genes after iCOX7B. N=3, *$p < 0.05$, ns, no significance.

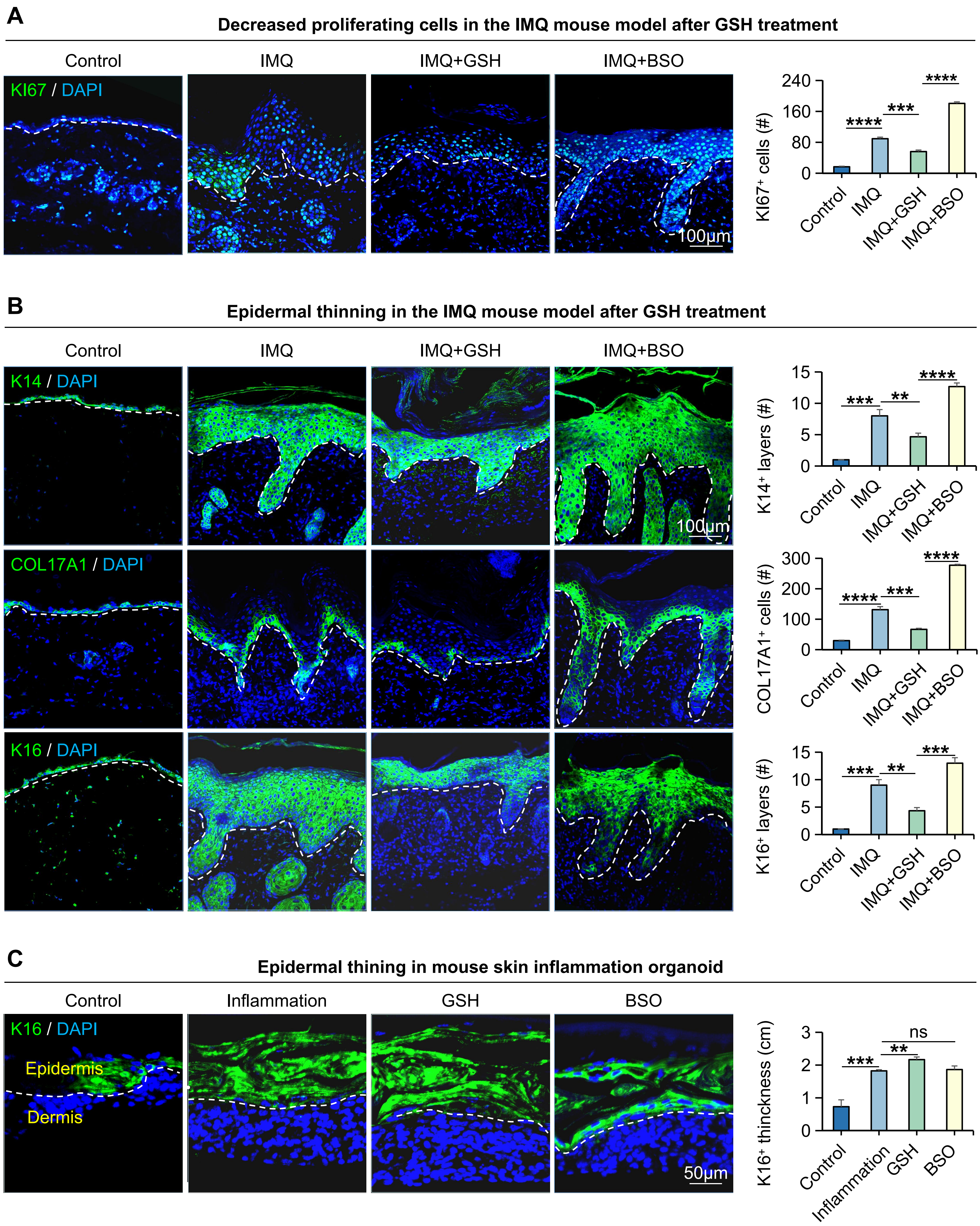
A
Decreased proliferating cells in the IMQ mouse model after GSH treatment
Control
IMQ
IMQ+GSH
IMQ+BSO
KI67 / DAPI
100μm
KI67⁺ cells (#)
240
160
80
0
****
***
****
Control
IMQ
IMQ+GSH
IMQ+BSO
B
Epidermal thinning in the IMQ mouse model after GSH treatment
Control
IMQ
IMQ+GSH
IMQ+BSO
K14 / DAPI
100μm
COL17A1 / DAPI
K16 / DAPI
K14⁺ layers (#)
15
10
5
0
***
**
****
COL17A1⁺ cells (#)
300
200
100
0
****
***
****
K16⁺ layers (#)
15
10
5
0
***
**
***
C
Epidermal thining in mouse skin inflammation organoid
Control
Inflammation
GSH
BSO
K16 / DAPI
Epidermis
Dermis
50μm
K16⁺ thinckness (cm)
3
2
1
0
***
**
ns
Control
Inflammation
GSH
BSO

Figure S6. Glutathione metabolism regulates changes in the thickness of the epidermis in IMQ-induced psoriasis-like mice.

A. Immunofluorescence staining of KI67 shows proliferation of epidermal cells in IMQ-induced psoriasis-like mice after modulation of glutathione metabolism and statistical analysis. Scale bar, 100 μm. N=3, ****$p < 0.0001$, ***$p < 0.001$.

B. Immunofluorescence staining of K14, COL17A1, and K16 shows epidermal thickness in IMQ-induced psoriasis-like mice after modulation of glutathione metabolism and statistical analysis. Scale bar, 100 μm. N=3, ****$p < 0.0001$, ***$p < 0.001$, **$p < 0.01$.

C. Immunofluorescence staining of K16 shows epidermal thickness and statistical analysis of inflammatory skin organoids. Scale bar, 50 μm. N=3, ***$p < 0.001$, **$p < 0.01$, and ns no significant change.

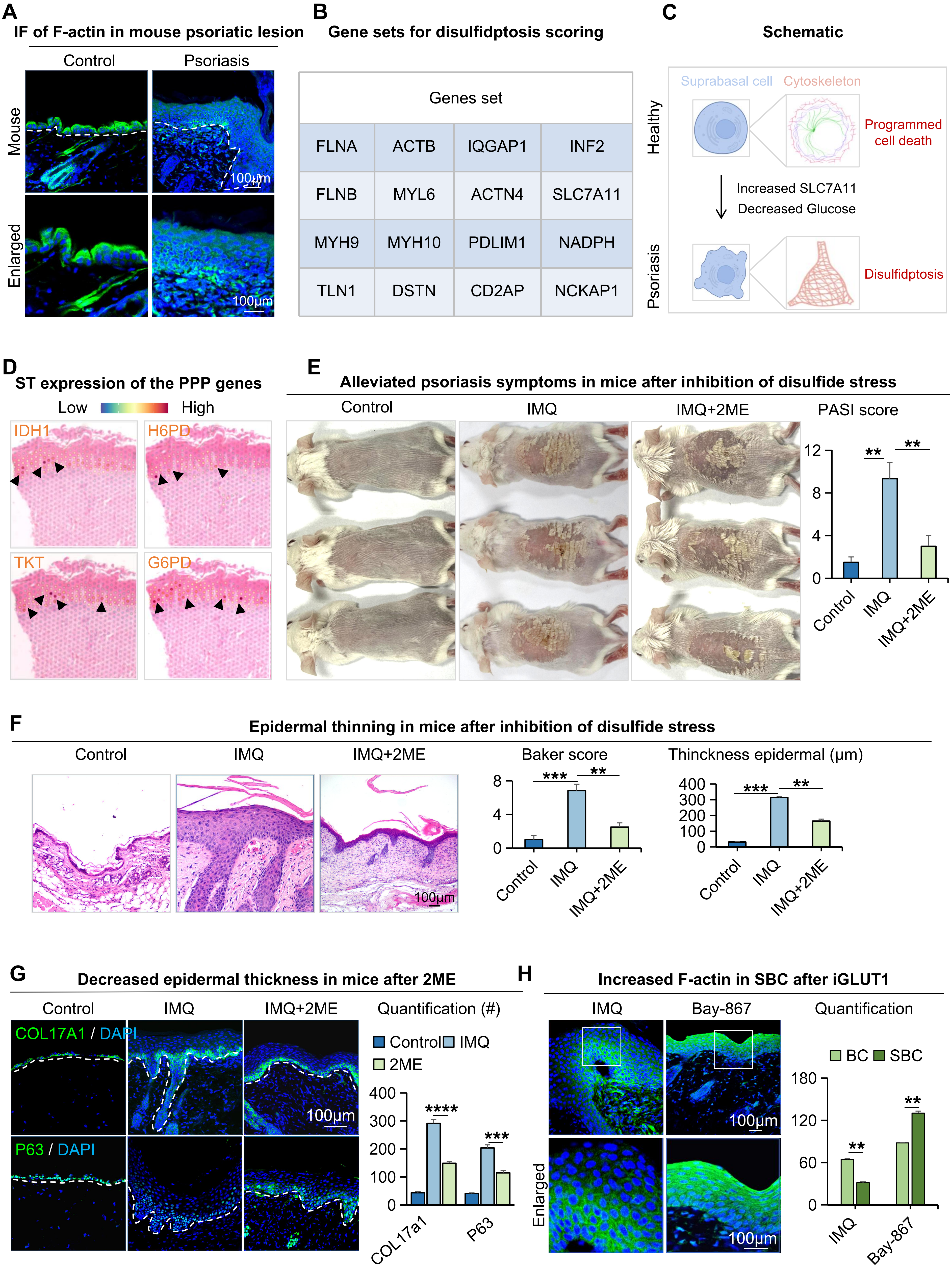
A
IF of F-actin in mouse psoriatic lesion
Control
Psoriasis
Mouse
Enlarged
100μm
100μm
B
Gene sets for disulfidptosis scoring
Genes set
FLNA ACTB IQGAP1 INF2
FLNB MYL6 ACTN4 SLC7A11
MYH9 MYH10 PDLIM1 NADPH
TLN1 DSTN CD2AP NCKAP1
C
Schematic
Suprabasal cell
Cytoskeleton
Healthy
Programmed cell death
Increased SLC7A11
Decreased Glucose
Psoriasis
Disulfidptosis
D
ST expression of the PPP genes
Low
High
IDH1
H6PD
TKT
G6PD
E
Alleviated psoriasis symptoms in mice after inhibition of disulfide stress
Control
IMQ
IMQ+2ME
PASI score
12
8
4
0
**
**
Control
IMQ
IMQ+2ME
F
Epidermal thinning in mice after inhibition of disulfide stress
Control
IMQ
IMQ+2ME
100μm
Baker score
8
4
0
***
**
Control
IMQ
IMQ+2ME
Thinckness epidermal (μm)
400
300
200
100
0
***
**
Control
IMQ
IMQ+2ME
G
Decreased epidermal thickness in mice after 2ME
Control
IMQ
IMQ+2ME
COL17A1 / DAPI
P63 / DAPI
100μm
Quantification (#)
Control
IMQ
2ME
400
300
200
100
0
****
***
COL17a1
P63
H
Increased F-actin in SBC after iGLUT1
IMQ
Bay-867
Enlarged
100μm
100μm
Quantification
BC
SBC
180
120
60
0
**
**
IMQ
Bay-867

Figure S7. Thinning of the epidermis layer in IMQ-induced psoriasis-like mice after 2ME treatment.

A. Immunofluorescence staining of COL17A1, P63, E-cadherin, and K16 shows changes in epidermal thickness in the IMQ-induced psoriasis-like mice. Scale bar, 100 μm. N=3, ****$p < 0.0001$, ***$p < 0.001$, **$p < 0.01$, *$p < 0.05$.

B. Gene sets for disulfidptosis scoring.

C. Schematic of disulfide death in psoriatic suprabasal cells.

D. Spatial transcriptomic data showing the location of PPP key genes expressed in psoriasis. PPP: pentose phosphate pathway.

E. Dorsal macroscopic features and PASI scores of mice in control, IMQ, and IMQ+2ME groups. 2ME: 2-Methoxyestradiol, inhibitors of disulphide reduction. N=3, **$p < 0.01$.

F. Representative H&E staining and baker scores of the back of mice in the control, IMQ, and IMQ+2ME groups; Statistical analysis of epidermal thickness. scale bar, 100 μm. N=3, ***$p < 0.001$, **$p < 0.01$.

G. Immunofluorescence staining of COL17A1 and P63 shows changes in epidermal thickness in the IMQ-induced psoriasis-like mice. Scale bar, 100 μm. N=3, ****$p < 0.0001$, ***$p < 0.001$.

H. Immunofluorescence staining shows increased expression of F-actin in suprabasal cells after iGLUT1. Scale bar, 100 μm. N=3, **$p < 0.01$.

Supplementary Table S1. List of primers used for qRT-PCR.

Species	Application	*Gene*	Forward primer (5'-3')	Reverse primer (5'-3')
human	qRT-PCR	*ATP5MC1*	TGTGAAGAGGACAATACCAGCG	CCAGCTTGTAATGGGCTCCAC
human	qRT-PCR	*COX6C*	CCAAAACCTCGGATGCGTG	AAATCTGCGTATGCCTTCTTTCT
human	qRT-PCR	*COX7B*	CTTGGTCAAAAGCGCACTAAATC	AAAATCAGGTGTACGTTTCTGGT
human	qRT-PCR	*GAPDH*	TGGCCTTCCGTGTTCCTAC	GAGTTGCTGTTGAAGTCGCA
human	qRT-PCR	*GSTA1*	CTGCCCGTATGTCCACCTG	AGCTCCTCGACGTAGTAGAGA
human	qRT-PCR	*GPX3*	GAGCTTGCACCATTCGGTCT	GGGTAGGAAGGATCTCTGAGTTC
human	qRT-PCR	*GCLM*	CATTTACAGCCTTACTGGGAGG	ATGCAGTCAAATCTGGTGGCA
human	qRT-PCR	*GLUT1*	TCCACCCGATGGACAGAATTG	GTAGGTGCCTGACACCGAC
human	qRT-PCR	*NDUFA13*	GGCCCATCGACTACAAACGG	CGCTCACGGTTCCACTTCATT
human	qRT-PCR	*NDUFB3*	GCTGGCTGCAAAAGGGCTA	CTCCTACAGCTACCACAAATGC
human	qRT-PCR	*NDUFS5*	TGCACATGGAATCGGTTATACTC	CCGAAGCAAACACTCTACGAAAT
human	qRT-PCR	*NDUFS6*	TTCGGTTTGTAGGTCGTCAGA	CCATCGCACGCTATCACCC
human	qRT-PCR	*NDUFS8*	CCATCAACTACCCGTTCGAGA	CCGCAGTAGATGCACTTGG
human	qRT-PCR	*SLC7A11*	GGTCCATTACCAGCTTTTGTACG	AATGTAGCGTCCAAATGCCAG
human	qRT-PCR	*UQCR10*	ATCGTGGGCGTCATGTTCTTC	ATGTGGTCGTAGATAGCGTCC
human	qRT-PCR	*UQCRC1*	GGGAGTGTGGATTGATGTTGG	TGTTCCCTTGAAAGCCAGATG
human	qRT-PCR	*UQCRQ*	CGCGAGTTTGGGAATCTGAC	TAGTGAAGACGTGCGGATAGG
mouse	qRT-PCR	*IL6*	TAGTCCTTCCTACCCCAATTTCC	TAGTCCTTCCTACCCCAATTTCC
mouse	qRT-PCR	*CXCL10*	CCAAGTGCTGCCGTCATTTTC	GGCTCGCAGGGATGATTTCAA
mouse	qRT-PCR	*CCL20*	GCCTCTCGTACATACAGACGC	CCAGTTCTGCTTTGGATCAGC
mouse	qRT-PCR	*S100A9*	ATACTCTAGGAAGGAAGGACACC	TCCATGATGTCATTTATGAGGGC
mouse	qRT-PCR	*IL1β*	GCAACTGTTCCTGAACTCAACT	ATCTTTTGGGGTCCGTCAACT

Supplementary Table S2. List antibodies uesd in this study.

Antibody	Isotype	Company	Cat #
ATP5MC1	Mouse	Abcam	ab119686
GAPDH	Mouse	Proteintech	60004-1-Ig
β-ACTIN	Mouse	Abcam	ab8226
BrdU	Mouse	Chemicon	MAB3222
Collagen XVII	Rabbit	Beyotime	AF1078
COX7B	Rabbit	Abcam	ab140629
E-cadherin	Rabbit	Proteintech	20874-1-AP
ENO1	Rabbit	Proteintech	11204-1-AP
F-actin	Mouse	Abcam	ab205
GCLC	Rabbit	Affinity	DF8550
GLUT1	Rabbit	Beyotime	AF1015
GPI	Rabbit	Proteintech	15171-1-AP
GSS	Rabbit	Beyotime	AF7037
KRT14	Rabbit	Boster	A01432
KRT16	Rabbit	Affinity	AF5482
KRT17	Rabbit	Bioss	bs-1431R
KI67	Rabbit	Proteintech	27309-1-AP
KLK7	Rabbit	Abcam	ab244367
LDHA	Rabbit	Affinity	DF6280
NDUFS6	Rabbit	Affinity	DF9671
NDUFS8	Rabbit	Abcam	ab249605
P63	Rabbit	GeneTex	GTX102425
P-Cadherin	Goat	R&D	AF761
PCNA	Rabbit	Affinity	AF0239
PFKP	Rabbit	Affinity	DF3234
SLC7A11	Rabbit	Proteintech	26864-1-AP
FITC anti-mouse CD3		4a Bioteche	FMA003-01-025
APC anti-mouse CD45		4a Bioteche	FMA045-01-025

Supplementary Materials and Methods

Real-time quantitative reverse transcription PCR (qRT-PCR)

The dorsal skin of IMQ-induced psoriasis-like mice was collected according to the time points of the experimental design and the tissue (human and mouse) was ground in liquid nitrogen. Total RNA was extracted from the tissues using TRIZOL reagent (#NR0002, Leagene, China), and the RNA was then reversed to cDNA using the ReverTra Ace RT-qPCR kit (#RR047Q, Takara, Japan) according to the manufacturer's protocols. gene expression was analysed using SYBR Green PCR Master Mix (#RR820A, Takara, Japan); GAPDH was used as an internal reference, and the primers are shown in table S1.

Hematoxylin and eosin (H&E) staining

Mouse back skin tissue was fixed in 4% paraformaldehyde solution(PFA, #P804537, Macklin, China) for 48 hours at 4℃, then was dehydrated, embedded in paraffin, and sectioned to obtain tissue samples. Samples were stained with hematoxylin and eosin at room temperature for 2 minutes, differentiated solution for 1 minute, then soaked in tap water for 5 minutes, dehydrated, xylene, and mounted with neutral resin. Finally, histopathological examination was performed by a phase-contrast microscope(Mito, China) and photographed with NScope 2.0.

Western blot

Total protein was extracted by grinding the tissue and prepared for Western blot as in the previous study [1], namely SDS-polyacrylamide gel electrophoresis before transfer to PVDF membrane (Millipore, Billerica, MA, USA). The membrane was then blocked with 5% milk and incubated with primary antibody overnight at 4˚C. The membrane was then washed three times with TBST, each time for 15 minutes, and the secondary antibody was incubated for 1 hour at 37˚C. The membrane was then visualized using the ECL reagent (Thermo Fisher, USA).

Flow cytometry

Firstly, the skin tissue was washed 3 times with pbs, the tissue was placed in a petri dish, 1ml of trypsin digest containing collagenase was added and the tissue was cut as much as possible with ophthalmic scissors. Put the dish into the incubator at 37 degrees , 60 minutes, blowing every 10min. Aspirate the supernatant, filter through a 100 mesh sieve, add 1ml of termination solution, transfer to a 15ml centrifuge tube at 1500rpm for 3min and discard the supernatant. Next, add 1ml pbs wash 3 times, 1500rpm 3min, discard the supernatant. Add 100ul flow staining solution and resuspend the cells, meanwhile, add 5ul CD4 and 5ul CD45 antibody, shake and mix well. Stain for 30 min at room temperature. Add 1ml of pbs and wash 3 times at 1500rpm for 3min, discard the supernatant, add 100ul of flow-through staining solution and prepare for testing.

Immunofluorescence staining

The paraffin-embedded samples were cut into 10um slices which were dried in an oven at 67 ˚ C for 30 minutes, then dewaxed in xylene, followed by gradient alcohol hydration, and antigen retrieval with citric acid (# C805019, Macklin, China) and sodium citrate solutions (#S818273, Macklin, China). Then, the slices with immunohistochemical strokes were blocked with 2% bovine serum albumin solution (BSA; #A8020, Solarbio, China) in an oven at 37 ˚ C for 1 hour, and then incubated with the primary antibody overnight at 4 ˚ C. The samples were rewarmed at room temperature for 2 hours, and after washing excess primary antibody, incubated with fluorescently-labeled secondary antibody (Alexa Fluor 488-conjugated goat anti-mouse IgG, Beyotime, China or 488-conjugated goat anti-mouse IgG, Beyotime, China) at 37 ˚ C for 2 hours. After wash, the samples were incubated with DAPI (#c-1002, Beyotime, China) at room temperature for 30 min, and finally mounted with an anti-fluorescent extractant. The image was taken under a laser confocal microscope (Lecia, Germany) at the Analysis and Testing Center of Chongqing University.

[1] Xu, K., Shao, Y., Xia, Y., Qian, Y., Jiang, N., Liu, X., Yang, L., and Wang, C. (2021). Tenascin-C regulates migration of SOX10 tendon stem cells via integrin-α9 for promoting patellar tendon remodeling. Biofactors 47, 768-777.
